# Mitochondrial calcium shapes B cell signaling and mitochondrial function

**DOI:** 10.3389/fimmu.2025.1710128

**Published:** 2025-12-11

**Authors:** Hakan Taskiran, Elena Czeslik, Leonhard Stark, Kathrin Kläsener, Theresa Elisa Schnalzger, Jürgen Ruland, Michael Reth, Julia Jellusova

**Affiliations:** 1 Institute of Clinical Chemistry and Pathobiochemistry, School of Medicine and Health, University Hospital of Technical University of Munich, Munich, Germany; 2 TranslaTUM, Center for Translational Cancer Research, Technical University of Munich, Munich, Germany; 3 Faculty of Biology, University of Freiburg, Freiburg, Germany; 4 International Max Planck Research School of Immunobiology, Epigenetics and Metabolism (IMPRS-IEM), Freiburg, Germany; 5 Department of Dermatology, School of Medicine and Health, Technical University of Munich, Munich, Germany; 6 Department of Rheumatology and Clinical Immunology, University Medical Center Freiburg, Centre for Biological Signalling Studies and Centre for Integrative Biological Signalling Studies, University of Freiburg, Freiburg, Germany

**Keywords:** B lymphocyte, calcium, signaling, metabolism, mitochondria

## Abstract

**Introduction:**

Calcium (Ca^2+^) signaling plays a pivotal role in determining B cell fate, shaping processes such as activation, differentiation, anergy or apoptosis. Upon B cell antigen receptor activation, Ca^2+^ is rapidly mobilized from the endoplasmic reticulum and supplemented by Ca^2+^ influx from the extracellular space, ultimately driving activation of various signaling pathways required for appropriate B cell responses. Although mitochondria also harbor significant levels of Ca^2+^, how mitochondrial Ca^2+^ dynamics are regulated in B cells in response to activation or other cues remains unknown, as do the functional consequences of altered mitochondrial Ca^2+^ levels.

**Methods:**

Chemical dyes as well as a genetically encoded Ca^2+^ sensor with a mitochondrial targeting sequence were used to study mitochondrial Ca^2+^ dynamics in response to various stimuli. Proximity ligation assays were performed to assess interaction between mitochondria and the endoplasmic reticulum. Primary mouse B cells and the Burkitt lymphoma cell line Ramos were used to study functional consequences of the loss of the Mitochondrial Calcium Uniporter.

**Results:**

Here, we show that mitochondrial Ca^2+^ levels dynamically respond to cell activation, stress and metabolic cues and that mitochondrial Ca^2+^ uptake is largely dependent on the Mitochondrial Calcium Uniporter. Reduced mitochondrial Ca^2+^ uptake has a negative impact on mitochondrial activity and also affects cell signaling. These findings demonstrate that changes in mitochondrial Ca^2+^ contribute to shaping functional B cell responses.

**Discussion:**

The spatial and temporal dynamics of Ca^2+^ accumulation within distinct subcellular compartments, particularly the cytosol, endoplasmic reticulum and mitochondria, are essential for translating extracellular and intracellular signals into specific cellular outcomes. Our study provides new insights into the regulation of Ca^2+^ homeostasis in B cells.

## 1 Introduction

Ca^2+^ is a ubiquitous second messenger regulating diverse cellular functions. In B cells, B cell antigen receptor (BCR) engagement triggers cytosolic Ca^2+^ accumulation (1). Ca^2+^ activates crucial signaling pathways, thereby governing cell fate decisions (1). Total cytosolic Ca^2+^ levels are shaped by the sum of Ca^2+^ influx from extracellular space via plasma membrane localized Ca^2+^ pumps and the release from intracellular Ca^2+^ stores (1). The endoplasmic reticulum (ER) and the mitochondria represent the main intracellular Ca^2+^ stores. While it is well understood that BCR activation induces Ca^2+^ release from the ER, the dynamics of mitochondrial Ca^2+^ (mCa^2+^) and their role in regulating B cell function remains comparatively underexplored. Mitochondria not only serve as metabolic hubs but also act as dynamic Ca^2+^ buffers (2). In other cell types, Ca^2+^-dependent mitochondrial enzymes have been reported to shape tricarboxylic acid (TCA) cycle activity as well as electron transport chain (ETC) function (1). Moreover, by releasing or sequestering Ca^2+^ from the cytosol, mitochondria can modulate cytosolic Ca^2+^ transients, thereby directing cell fate decisions. The involvement of mCa²^+^ in regulating cytosolic signaling and cell fate decisions has been demonstrated across various cell types, including lymphocytes (3–5). In addition to directly binding to Ca^2+^ dependent signaling molecules, mCa^2+^ can also affect signal transduction via modulating redox balance. The production of reactive oxygen species (ROS) is a natural side product of mitochondrial oxidative metabolism. Mitochondrial ROS contributes to signal transduction upon BCR signaling (6). However, excess production of mitochondrial ROS inhibits BCR signaling (7). Mitochondrial Ca^2+^ can change redox balance by boosting mitochondrial activity, but also by triggering mitochondrial damage (8, 9) thereby acting as both a physiological and pathological effector depending on the signaling context.

Mitochondrial Ca^2+^ levels are determined by the balance between mCa^2+^ uptake and release. Ca^2+^ ions enter the mitochondrial matrix through a channel known as the mitochondrial Ca^2+^ uniporter (MCU). The MCU oligomers form a pore, which is highly selective for Ca^2+^ but displays a rather low affinity for its target (10). Due to this low affinity, mCa^2+^ uptake occurs primarily at sites of high Ca^2+^ concentrations such as the ER-mitochondria contact sites (11). These contact sites are highly organized platforms that facilitate the exchange of lipids and other small molecules in addition to Ca^2+^ (10). The formation of new ER-mitochondria contact sites is dynamic and represents a mechanism by which cells support ER-mitochondria communication and enhance mCa^2+^ uptake. An additional layer of regulation of mCa²^+^ uptake can be achieved through direct modulation of MCU channel activity (12). The MCU pore is part of a multiprotein complex and interacts with various regulators including mitochondrial calcium uniporter regulator 1 (MCUR1), the mitochondria Ca^2+^ uptake proteins 1,2,3 (MICU 1,2,3) and the essential MCU regulator (EMRE). These regulators help to fine tune mCa^2+^ uptake and the exact composition of the complex can vary between tissues and contributes to tissue specific activity of MCU (12–14). In summary cells possess a multitude of means to control mCa^2+^ levels and utilize them to respond to cellular activation, ER-stress or other physiologically meaningful conditions (15, 16). The dynamics of mCa^2+^ uptake in B cells in response to various stimuli is currently largely unknown. Moreover, the functional consequences to changes in mCa^2+^ levels are currently poorly understood.

A recent model proposes that Ca^2+^ homeostasis plays a central role in how B cells respond to BCR activation (17). Upon BCR stimulation, B cells are activated and seek T cell help to initiate a germinal center response. The ultimate goal of the germinal center response is the differentiation to memory B cells and plasma cells secreting high affinity antibodies. The need for a second signal for a full initiation of B cell immune responses is an important mechanism to safeguard against autoimmunity. While autoantigens can induce BCR signaling, the lack of co-stimulatory signals prevents the entry of these cells into the germinal center, ultimately resulting in their elimination. It has recently been shown, that BCR activation initially induces an increase in mitochondrial activity but ultimately leads to mitochondrial blebbing and cell death in the absence of costimulatory signals (17). It has been demonstrated that an increase in intracellular Ca^2+^ causes mitochondrial dysfunction if BCR-stimulated cells do not receive additional signals within a few hours after stimulation. However, the exact molecular mechanisms and the role of mCa^2+^ in this process remain unclear. Consequently, understanding how mCa^2+^ shapes B cell biology is essential, not only for advancing fundamental immunology, but also for uncovering potential therapeutic targets in B cell-related diseases.

Here we show that contrary to prevailing assumptions, mitochondrial depolarization or mCa^2+^ overload in B cells does not inherently trigger cell death. Instead, mCa^2+^ levels are dynamically regulated by BCR activation, ER stress, and the surrounding metabolic microenvironment. Notably, the mitochondrial Ca^2+^ uniporter (MCU) emerges as a key mediator, coordinating mitochondrial activity and cytosolic signaling pathways. These findings underscore a nuanced role for mCa^2+^ in B cell function, decoupling bioenergetic disruption from immediate apoptotic outcomes.

## 2 Materials and methods

### 2.1 Cell culture and genetical modification of Ramos cells

#### 2.1.1 Cell culture

The Burkitt lymphoma cell line Ramos expressing the murine retrovirus receptor mCAT1, was kindly provided by M. Reth. Ramos cells were cultured in RPMI-1640 medium with GlutaMAX (Thermo Scientific) supplemented with 10% heat inactivated fetal bovine serum (FBS) (Gibco), 100U/ml penicillin, 100µg/ml streptomycin (Gibco), 10mM HEPES (Gibco) and 50µM β-mercaptoethanol (Gibco) in a 5% CO_2_ atmosphere at 37°C.

For hypoxia experiments, Ramos cells were plated at 5x10^5^ cells/ml in RPMI-1640 media with GlutaMAX supplemented with 10% heat inactivated FBS, 100U/ml penicillin, 100µg/ml streptomycin, 10mM HEPES and 50µM β-mercaptoethanol in a 5% CO_2_ incubator in either 21% (normoxia) or 4.5% (hypoxia) oxygen at 37°C for 24 h.

Platinum-E (Plat-E) cells were cultured in RPMI-1640 media with GlutaMAX supplemented with 10% heat inactivated FBS, 100U/ml penicillin, 100µg/ml streptomycin, 10mM HEPES and 50µM β-mercaptoethanol in a 37°C incubator with 5% CO_2_.

#### 2.1.2 Gene deletion

CRISPR RNAs (Alt-R CRISPR-Cas9 crRNAs) targeting human MCU (Hs. Cas9.MCU.1.AB, TACACCAGAGGATCGCTTCC) and MCUR1 (Hs.Cas9.MCUR1.1. AB, GTCTTCCAGTAAGCACACTA) genes were selected from predesigned Alt-R CRISPR-Cas9 guide RNAs (IDT). Alt-R CRISPR-Cas9 genome editing system was used to knockout MCU and MCUR1 genes in Ramos B cells. Cell culture density was adjusted to a concentration of 5-7.5x10^5^ cells/ml one day before electroporation. After mixing Alt-R CRISPR-Cas9 crRNAs and tracrRNA (IDT) in equimolar concentration to a final duplex concentration of 44µM, oligo mix were heated at 95°C for 5 min and cooled down to room temperature (RT) to form guide RNAs (gRNAs). Ribonucleoprotein complexes were assembled by incubating 44µM crRNA:tracrRNA duplex and 37µM Alt-R Cas9 enzyme (IDT) for 20 min at RT. 4D-Nucleofector system (Lonza) was used for efficient transfection of B cell lines. Ramos B cells (5x10^5^-1x10^6^ cells) were resuspended in 20µl completed nucleofector solution per electroporation. After ribonucleoprotein complexes were mixed with cell suspension and 10.8µM Alt-R CRISPR-Cas9 Electroporation enhancer (IDT), the final mixtures were transferred into Nucleocuvette vessels. The transfected cells were resuspended and cultured in pre-warmed recovery medium (RPMI-1640 media with GlutaMAX supplemented with 20% heat inactivated FBS and 10mM HEPES). After 36 h, single cell clones were generated (0.5-0.9 cells/well, 96-well plate).

#### 2.1.3 Transduction

To generate the pMIG-mGCaMP6s construct, pMIG empty vector was linearized using HindIII and EcoRI restriction enzymes. Duplicated mitochondrial targeting sequence (MTS) and GCaMP6s were amplified and inserted to linearized pMIG empty vector via In-Fusion HD cloning system (Takara).To generate the pMIG-mGCaMP6s_mKO2 construct a viral cleavage peptide followed by a red fluorescent protein (P2A-mKO2) sequence was inserted into pMIG-mGCaMP6s plasmid using In-Fusion HD cloning kit. The MTS originates from the subunit VIII of human cytochrome c oxidase (18, 19) and has the following sequence:

atgtccgtcctgacgccgctgctgctgcggggcttgacaggctcggcccggcggctcccagtgccgcgcgccaagatccattcgttgggggatctgtccgtcctgacgccgctgctgctgcggggcttgacaggctcggcccggcggctcccagtgccgcgcgccaagatccattcgttgggggat

To generate the MSCVpuro-MCU cDNA construct, MSCVpuro empty vector was linearized using XhoI and EcoRI restriction enzymes. Total RNA from Ramos B cells was extracted using Quick-RNA MicroPrep kit (Zymo Research). The cDNA was synthesized using SCRIPT Reverse transcriptase (Jena) and Oligo (dT)18 primer (Thermo Scientific). MCU cDNA was amplified with primers containing XhoI and EcoRI cutting sites. Later, PCR amplicon was digested with these two restriction enzymes and inserted into the linearized MSCVpuro empty vector backbone.

The retroviral plasmids encoding mGCaMP6s, mGCaMP6s_mKO2 and MCU cDNA were transfected along with pKat plasmid encoding a viral coat protein into Platinum-E ecotropic retroviral packaging cell line using PolyJet transfection reagent (SignaGen). The virus containing supernatant was collected after 48 and 72 h and filtered through a 0.2µm filter.

Ramos cells were transduced with mGCaMP6s or mGCaMP6s_mKO2 retrovirus supernatants in the presence of 10µg/ml Polybrene reagent (Merk Millipore) for 4 h at 37°C with 5% CO_2_. The cells expressing mGCaMP6s and mGCaMP6s_mKO2 were sorted for EGFP signal and mKO2 signal, respectively.

Ramos WT and MCU^-/-^ cells were transduced with MSCVpuro or MSCVpuro-MCU cDNA retrovirus supernatants, which were pre-diluted 1/2 in culture media, in the presence of 10µg/ml Polybrene reagent for 4 h at 37°C with 5% CO_2_. Treatment with 2µg/ml puromycin (InvivoGen) for 3 days was used to select transduced cells.

### 2.2 Generation and cell culture of *Mcu^-/-^* mouse B cells

#### 2.2.1 Animals

*Mcu^fl/fl^* mice (B6;129S-*Mcu^tm1.1Jmol^*/J, The Jackson Laboratory) were crossed to *mb1-CreER^T2^* mice (B6.C-*Cd79a^tm3(cre/ERT2)Reth^*/EhobJ, The Jackson Laboratory). The resulting *Mcu^fl/fl^ x mb1^CreERT2^* mice express a tamoxifen-inducible form of Cre recombinase under the control of the mb-1 promoter. Both male and female mice between 8–15 weeks of age were used for experiments. All mice were housed under specific pathogen free conditions. Mice were sacrificed under deep isoflurane anesthesia (max. 5% volume (v/v), inhalation) followed by cervical dislocation. Animal experiments were carried out in accordance with the German Animal Welfare Act and approved by the regional council (Regierung von Oberbayern).

#### 2.2.2 B cell isolation and culture

Total splenic cell suspensions were used to obtain B cells using MojoSort mouse pan B cells isolation kit II (BioLegend). Primary B cells were plated at 2x10^6^ cells/ml in RPMI-1640 media with GlutaMAX supplemented with 10% FBS, 100U/ml penicillin, 10µg/ml streptomycin, 2mM L-glutamine (Gibco), 1mM sodium pyruvate (Gibco), 1X MEM non-essential amino acids solution (MEM NEAA, Gibco), 10mM HEPES, 50µM β-mercaptoethanol and cultured at 37°C with 5% CO_2_. For anti-IgM stimulation, freshly isolated mouse B cells (2x10^6^ cells/ml) were stimulated in complete mouse media with 10µg/ml anti-mouse IgM (Jackson ImmunoResearch) for indicated times.

#### 2.2.3 *Mcu* deletion using tamoxifen

*Mcu^fl/fl^ x mb1^CreERT2^*and control mice were injected with 100µl of 10mg/ml tamoxifen + 10% ethanol (Sigma-Aldrich) in olive oil (Sigma Aldrich), for three consecutive days to induce Cre recombinase activity. The group of control mice consisted of *Mcu^fl/fl^ x mb1^wt^* and *Mcu^wt/wt^x mb1^CreERT2^*mice. No differences were observed between the two genotypes. Mice were sacrificed 8 days after tamoxifen injection.

#### 2.2.4 *Mcu* deletion using TAT-CRE

TAT-CRE (Sigma Aldrich) was used to induce deletion of MCU gene in *Mcu^fl/fl^* B cells. *Mcu^wt/wt^* mice were used as controls and treated the same as the experimental mice. Freshly isolated mouse B cells were resuspended in OPTIMEM (1X) with GlutaMAX (Thermo Scientific) + TAT-CRE (3-4µM, Sigma Aldrich) and incubated in a 37°C incubator for 1 h. The cells were washed with PBS + 10% FBS and plated at 2x10^6^ cells/ml in RPMI-1640 media with GlutaMAX supplemented with 10% FBS, 100U/ml penicillin, 10µg/ml streptomycin, 2mM L-Glutamine, 1mM sodium pyruvate, 1X MEM NEAA, 10mM HEPES, 50µM β-mercaptoethanol and 10µg/ml anti-mouse IgM. The cells were cultured at 37°C with 5% CO_2_ for the indicated time.

#### 2.2.5 Analysis of *Mcu* gene deletion efficiency

To assess gene deletion efficiency splenic B cells isolated from MCU^-/-^ and control animals were genotyped using Phire Tissue Direct PCR Master mix. Primers used: forward primer *5’-AGGTGATGATAGTGAAGGTGTATG-3’;* reverse primer *5’-GTGGTGGAGCACATGAGTAA-3’*; reverse primer 2 *5’-TCTGGAGGAGAGAGGTTTGT-3’*. Forward primer and reverse primer 2 detect the *Mcu* gene prior to Cre-mediated recombination (Mcu-wt-allele: 952 bp; Mcu-fl-allele: ~1000bp). Forward primer and reverse primer detect the *Mcu* gene after recombination (the deleted Mcu-fl-allele: ~1250bp).

### 2.3 Cell signaling analysis via immunoblots

For the analysis of cell signaling minimal or complete media was used. Minimal media consisted of PBS with 0.5% FBS, 5.55mM D-Glucose, 2mM MgCl_2_ and 0.5mM CaCl_2_. Complete media was composed of RPMI-1640 with GlutaMAX supplemented with 10% FBS, 100U/ml penicillin, 100µg/ml streptomycin, 2mM L-glutamine (Gibco), 1mM sodium pyruvate (Gibco), 1X MEM non-essential amino acids solution (MEM NEAA, Gibco), 10mM HEPES, 50µM β-mercaptoethanol.

To assess BCR-mediated B cell activation, B cells were first washed with minimal media. Cell pellets and anti-IgM solutions were incubated at 37°C for 5 min. B cells were incubated in anti-IgM solution for indicated times and then washed with ice-cold PBS to stop the reaction. Cells were pelleted, washed with PBS and lysed using 4% SDS lysis buffer supplemented with protease and phosphatase inhibitors (62.5mM Tris-HCl pH 6.8 (Sigma-Aldrich), 10% glycerol (Sigma-Aldrich), 4% SDS (Invitrogen), 1X protease inhibitor cocktail (Sigma-Aldrich), 20mM NaF (Carl Roth) and 1mM Na_3_VO_4_ (Sigma-Aldrich)) at 95°C for 10 min.

To understand the role of FCCP in BCR-mediated B cell activation, cells were washed with minimal media or complete mouse media and incubated at 37°C for 5 min, mouse B cells (3x10^6^ cells) were stimulated with 10µg/ml anti-mouse IgM or 2µM FCCP (Sigma-Aldrich) or the combination of both for indicated time periods. For FCCP pre-treatment experiments, mouse B cells (3x10^6^ cells) were washed with complete mouse media and heated at 37°C for 5 min. The cells were first treated with pre-heated FCCP (2µM) or DMSO for 5 min at 37°C and stimulated with pre-heated anti-mouse IgM (10µg/ml) for indicated time points. The cells were further processed as described above.

For rapamycin, ibrutinib and LY294002 control immunoblots, Ramos B cells (8x10^5^ cells) were treated with rapamycin (100nM, Sigma-Aldrich), ibrutinib (2µM, LKT laboratories) or LY294002 (10-25μM, Invitrogen) for 4 h in a 37°C incubator before anti-human IgM stimulation (5µg/ml, Southern Biotech) in minimal media and lysate preparation.

Protein concentrations were determined using the BCA protein assay kit (Thermo Scientific). 10µg total cell lysates were mixed with loading buffer (5X: 250mM Tris-HCl pH 6.8, 6.25% glycerol, 1% β-mercaptoethanol, 5% SDS, 0.1% bromophenol blue) and then heated at 95°C for 10 min. Lysates were separated using hand cast BIS/TRIS acrylamide gels in Mini-PROTEAN Tetra cells (Bio-Rad) and later transferred to 100% ethanol activated Polyvinylidene Fluoride (PVDF) membranes (Carl Roth) using Mini Trans-Blot cell (Bio-Rad). Membranes were blocked for 1h in 5% BSA (Capricorn Scientific) in tris-buffered saline with Tween 20 (0.1%, Sigma-Aldrich). Membranes were probed with antibodies against MCU, MCUR1, NFAT2, total and cleaved caspase 3, pPLCγ2(Y1217), pBtk(Y223), pS6(S235/236), pAkt(S473), PLCγ2, Btk, Akt (Cell Signaling Technology), and β-actin (Proteintech) overnight at 4°C. Blots were stained with appropriate secondary antibodies and ChemiDoc MP imaging system (Bio-Rad) was used for visualization.

### 2.4 Analysis of cell survival, proliferation and redox balance via flow cytometry

For cell proliferation assays, Ramos cells were loaded with eFluor 670 (2.5µM, Invitrogen) for 6 min at 37°C and washed with complete media for 5 min. The cells were seeded on 96-well plates (5x10^4^ cells/well). The same staining protocol was applied to mouse B cells. Stained mouse B cells were seeded on 96-well plates (2x10^5^ cells/well) and were cultured with anti-mouse IgM (10µg/ml) or anti-mouse CD40 (0.2µg/ml, HM40-3, Santa Cruz Biotechnology) + IL4 (10ng/ml, Sigma-Aldrich) for 3 days. For FCCP experiments, in addition to BCR stimulation, plated cells were cultured in the presence or absence of FCCP (1-2µM) for 3 days. DMSO was used as a control. For FCCP pre-treatment experiment, mouse B cells were pre-treated with FCCP (2µM) or DMSO for 5 min and later cultured with anti-mouse IgM (10µg/ml) for 3 days. To calculate the average number of cell divisions the logarithm with the base two was calculated from the obtained mean fluorescence intensity of unstimulated cells and stimulated cells. The difference between these two values represents the average number of cell divisions.

To assess cell survival, FCCP-treated (1-2µM) B cells were stained with Zombie Aqua, a fixable viability dye (1/1000, BioLegend) in PBS for 10 min at 4°C. The cells were washed and measured in FACS buffer (PBS + 2% FBS). Forward scatter (FSC) versus side scatter (SSC) density properties from cells used in the above-described proliferation experiments were analyzed to determine the percentage of living cells. 20K FSC-A threshold was set to remove cell debris. Annexin V and propidium iodide (PI) staining was performed using APC Annexin V apoptosis detection kit with PI (BioLegend) according to the manufacturer’s instructions.

For measurement of ER mass, Ramos and mouse B cells (2.5-5x10^5^ cells) were stained in HBSS with ER-Tracker Blue-White DPX (1µM, Invitrogen) for 15 min at 37°C. After washing twice with HBSS, the cells were measured.

For mitochondrial mass staining, Ramos and mouse B cells (2.5-5x10^5^ cells) were stained with MitoBright LT deep red (0.1µmol/L, 15 min, Dojindo) and MitoTracker green FM (30nM, 20 min, Invitrogen) in complete media at 37°C, respectively. Ramos B cells were washed twice with media and mouse B cells were washed twice with PBS before measurement.

For mitochondrial ROS measurement, Ramos B cells (2.5x10^5^ cells) were stained in HBSS with mtSOX deep red (10µmol/l, Dojindo) for 30 min at 37°C. The cells were washed twice in HBSS before measurement. NAC (500µM) was used as a control. For anti-IgM stimulation experiments, Ramos B cells (1x10^6^ cells) were stained with mtSOX deep red using the same protocol. Later, cell suspension was divided into two and pelleted. Before measurement, the cell pellets and anti-human IgM solution (5µg/ml, in HBSS) were incubated for 5 min at 37°C. Mitochondrial ROS levels were measured after 1 min of anti-IgM stimulation. To determine mitochondrial ROS levels of FCCP-treated mouse B cells, the cells (4x10^5^ cells) were stained in minimal media with MitoSOX red (5µM, Invitrogen) in the presence of FCCP (2µM) or DMSO for 10 min at 37°C. The cells were washed twice with minimal media containing FCCP (2µM) or DMSO and measured in these solutions. NAC (500µM) was used as a control.

To assess total ROS levels, B cells (4x10^5^ cells) were stained in minimal media with CellROX deep red (5µM, Invitrogen) in the presence of FCCP (2µM) or DMSO for 25 min at 37°C. The cells were washed three times with minimal media containing FCCP (2µM) or DMSO and resuspended in these solutions for measurement. NAC (500µM) was used a control. For FCCP addition experiments, the same staining protocol was performed without any treatment and the cells were resuspended in minimal media with FCCP (2µM) or DMSO right before measurement. Total ROS levels were measured at 1 and 5 min. The same protocols were also carried out using complete media.

### 2.5 Cytosolic and mitochondrial Ca^2+^ measurements

#### 2.5.1 Ca^2+^ measurement in Ramos and mouse B cells

B cells (3x10^6^ cells) were loaded with 3µM Indo-1 AM (AAT Bioquest) in FACS buffer for 30 min at 37°C. Loaded cells were washed with FACS buffer and resuspended in minimal media. The cell suspension was kept on ice and in the dark. Before measurement, the cell suspension and the stimulating agents were pre-warmed at 37°C for 5 min. Anti-IgM or FCCP was added after 25–30 seconds of baseline recording to a final concentration of 5-10ug/ml and 1-2uM, respectively. Ratiometric indo-1 measurement was performed using LSRFortessa (BD).

To measure total cytosolic Ca^2+^ levels, stimulated murine B cells (5x10^5^ cells) were loaded with 3µM Indo-1 AM in FACS buffer for 30 min at 37°C. Loaded cells were washed with FACS buffer and resuspended in minimal media. The cell suspension was kept on ice and in the dark. Ca^2+^-bound and free Indo-1 fluorescence was measured with an LSRFortessa (BD).

Ramos mGCaMP6s cells were used for mCa^2+^ measurements. The cells (2.5x10^5^ cells) were treated with FCCP (2µM) and BAPTA-AM (10µM, Sigma-Aldrich) for 30 min and kept as pellets. Anti-human IgM (5µg/ml) solution and the cell pellets were incubated at 37°C for 5 min. One min after resuspending the pellets in anti-human IgM solution, mCa2+ levels were measured. Ramos mGCaMP6s_mKO2 cells were used to perform ratiometric mCa2+ measurements. The same FCCP and BAPTA treatment protocol was followed using Ramos mitoGCaMP6s_mKO2 cells. MitoGCaMP6s/mKO2 ratio was measured to determine mCa2+ levels. To assess the effect of thapsigargin on mCa2+ levels, Ramos mGCaMP6s_mKO2 cells (2.5x10^5^ cells) were washed with minimal medium and then kept as a pellet. The cells were resuspended in minimal medium with thapsigargin (5µM, Sigma-Aldrich) or DMSO and incubated for 1 minute before measurement. Anti-human IgM stimulation was used as a control. To measure mitochondrial Ca^2+^ levels after nocodazole treatment, Ramos mGCaMP6s_mKO2 cells (7.5x10^5^ cells/ml) were treated in complete media with 10µM nocodazole in a 37°C incubator with 5% CO_2_ for 12h. To assess the effect of taurine on mCa^2+^ levels, the cells were either cultured for 24h in 2.5µM taurine or for 4h followed by anti-IgM stimulation for 5min.

To study the kinetics of mCa^2+^ flux the cells (1x10^6^ cells) were pre-treated with DMSO, rapamycin (100nM, Sigma-Aldrich), ibrutinib (2µM) or LY294002 (25μM) for 4 h at 37°C. The cells were washed and resuspended in minimal medium. The cell suspension was kept on ice and in the dark. Before measurement, the cell suspension and the stimulating agents were pre-warmed at 37°C for 5 min. Anti-IgM was added after 25–30 seconds of baseline measurement to a final concentration of 5µg/ml. MGCaMP6s/mKO2 ratio measurement was performed using LSRFortessa.

Ramos mGCaMP6s_mKO2 expressing cells were loaded with Indo-1 AM to perform simultaneous measurement of mitochondrial and intracellular Ca^2+^ dynamics. Before measurement, the cell suspension and stimulating agents were pre-warmed at 37°C for 5 min. Anti-IgM or FCCP was added after 25–30 seconds of baseline measurement to a final concentration of 5µg/ml and 2µM, respectively. Calcium bound/free Indo-1 and MitoGCaMP6s/mKO2 ratio measurements were performed using LSRFortessa (BD).

To assess mCa^2+^ levels, mouse B cells (3-5x10^5^ cells) were stained in complete mouse media with Rhod-2 AM (1µM, Invitrogen) for 20 min at 37°C. The cells were washed with complete media for 5 min at 37°C and kept as a pellet on ice and in the dark until measurement. BAPTA-AM was used as a control. To assess mCa^2+^ levels upon BCR stimulation, mouse B cells (2x10^6^ cells/ml) were stimulated in complete mouse media with anti-mouse IgM (10µg/ml) for 1 and 4 h. Stimulated or resting mouse B cells (5x10^5^ cells) were stained for mCa^2+^ in complete mouse media with or without anti-mouse IgM using the Rhod-2 AM staining protocol as described above. For anti-IgM stimulation experiments, mouse B cells (2x10^6^ cells) were stained in minimal medium with 1µM Rhod-2 AM for 20 min and then washed in minimal medium for 5 min at 37°C. The cell suspension was divided into two and pelleted. Before measurement, cell pellets and anti-mouse IgM solution (10µg/ml, in minimal medium) were warmed for 5 min at 37°C. Mitochondrial Ca^2+^ levels were measured after 1 and 5 min of BCR stimulation. Ramos B cells were stained for mCa^2+^ as described above and stimulated with anti-human IgM (5µg/ml) for a minute.

#### 2.5.2 Mitochondrial Ca^2+^ measurement in peripheral blood mononuclear cells

Healthy donor-derived blood samples were obtained from Bavarian Red Cross (Munich, Germany) and were isolated by density gradient centrifugation. In brief, the blood samples were diluted 1:3 with PBS and poured into a 50 ml falcon tube containing 15 ml Ficoll (Ficoll-Paque PLUS, Cytiva). After centrifugation (400g, 30 min, room temperature, no brakes), the buffy coat containing the PBMCs was carefully transferred into a new falcon and washed twice with PBS. Cells were cryopreserved in FBS + 10% DMSO at -80°C. The cells were thawed in cell culture medium (RPMI-1640 media with GlutaMAX supplemented with 10% heat inactivated fetal bovine serum (FBS) (Gibco), 100U/mL penicillin, 100µg/mL streptomycin (Gibco), 10mM HEPES (Gibco) and 50µM β-mercaptoethanol) and were rested in the incubator (5% CO_2_ atmosphere at 37°C) for a few hours before starting experiments. To assess mCa^2+^ levels in human B cells, PBMCs (1,5 – 2x10^6^ cells) were stained with Zombie Aqua (1/1000, BioLegend) in PBS for 10 min at 4°C to exclude dead cells. The cells were washed with PBS before subsequent staining with human anti-CD19 APC (1/100, BioLegend) in PBS + 2% FBS for 15 min at 4°C to identify the B cell population. Cells were washed twice with PBS + 2% FBS. PBMCs were then stained in minimal medium with Rhod-2 AM (1µM, Invitrogen) for 20 min at 37°C and then washed in minimal medium for 10 min at 37°C. The cell suspension was pelleted and kept on ice and in the dark until measurement. Before measurement, cell pellets and anti-human IgM solution (5 µg/mL, in minimal medium) were warmed for 5 min at 37°C. Mitochondrial Ca^2+^ levels were measured after 1 min of BCR stimulation. BAPTA-AM was used as a control.

### 2.6 Fluorescence intensity measurement of CellROX deep red

CellROX deep red working solution (5µM) was prepared in complete medium and plated on flat-bottom 96 well plates (200µl/well). FCCP, DMSO and hydrogen peroxide (H_2_O_2_) solutions that were prepared in complete medium were added to CellROX deep red working solution and incubated for 5 min at room temperature. Spark microplate reader (Tecan) was used to measure fluorescence intensity [Excitation wavelength: 620nm (20) and Emission wavelength: 680nm (30)]. Final FCCP and H_2_O_2_ working concentrations were 2µM and 0.24% (w/v, Sigma-Aldrich), respectively.

### 2.7 Cell growth measurement using the CCK8 kit

2 x 10^4^ cells in 100µl complete media were plated on day 0 on 3 identical plates. On the indicated days, 10µl of the CCK-8 solution (Sigma-Aldrich) were pipetted into each well of one plate. OD was measured at 450nm using the Multiskan FC optical reader (Thermo Fisher Scientific) after a 2 h of incubation at 37°C. The OD value from a background sample containing no cells was subtracted from all samples measured on the same plate.

### 2.8 Proximity ligation assays and confocal microscopy

Anti-VDAC1 (clone 20B12AF2, Abcam) and anti-IP3R1 (clone E-8, Santa Cruz Biotechnology) antibodies were used to generate proximity ligation assay (PLA) probes using Duolink *In Situ* Probemaker kit (Sigma-Aldrich). For Ramos cells, diagnostic slides (Thermo Scientific) were coated with poly-DL-lysine (0.1mg/ml, Sigma-Aldrich) for 2 h at 37°C and then washed with PBS three times. Ramos cells (1x10^5^ cells) or mouse B cells (3-4x10^5^ cells) were settled in PBS on diagnostic slides for 30 min at 37°C. Then, the cells were fixed with 4% formaldehyde (Thermo Scientific) in PBS for 15 min at RT, permeabilized with 0.5% saponin (Sigma-Aldrich) in PBS for 30 min (15 min at 4°C and 15 min at RT) and blocked with Duolink blocking solution (Sigma-Aldrich) for 1 hour at 37°C. The cells were later incubated in PBS with anti-VDAC1 (1/100) and anti-IP3R1 (1/100) PLA probes overnight at 4°C. PLA was performed according to the manufacturer’s instructions (Sigma-Aldrich). The resulting samples were mounted in Fluoromount-G with DAPI (Invitrogen). Fluorescence was detected by using an inverted confocal microscope, Leica TCS SP8 (Leica) with a 40x oil immersion objective. For each sample, at least 1,500 cells were imaged across multiple randomly selected regions. Quantification of detected signal per nucleus was performed by using CellProfiler Software (the Broad Institute of MIT and Harvard).

For nocodazole experiments, Ramos cells were treated with 10µM nocodazole for 12 h in a 37°C incubator. The cells were settled in PBS with 10µM nocodazole on poly-DL-lysine coated diagnostic slides at 37°C for 30 min. PLA was performed as described. DMSO was used as a control. For time-course anti-IgM stimulation experiments, Ramos cells and mouse B cells were settled in PBS on diagnostic slides for 30 min at 37°C and then stimulated in PBS with anti-IgM for the indicated time (5µg/ml anti-human IgM for Ramos cells and 10µg/ml for mouse B cells). For anti-CD40 + IL4 or LPS pre-stimulation experiments, freshly isolated mouse B cells (2-3x10^6^ cells/ml) were stimulated in complete mouse media with 5µg/ml anti-mouse CD40 (clone 1C10 (Thermo Scientific) or clone FGK45 (BioLegend)) + 10ng/ml IL4 or 5µg/ml LPS (Sigma-Aldrich) overnight. Later, pre-stimulated cells (3x10^5^ cells) were settled in PBS on diagnostic slides for 30 min at 37°C and further stimulated in PBS with 10µg/ml anti-mouse IgM for indicated times. After anti-IgM stimulation, the same PLA protocol was performed.

MitoBright LT deep red- and Rhod-2 AM-stained Ramos B cells were allowed to settle onto ibidiTreat coated cell culture slides (Ibidi). Experiments with live cells were performed using a Leica TCS SP8 microscope (Leica) with a 40x water immersion objective. All images were processed using ImageJ (NIH).

### 2.9 Metabolic flux analysis

A Seahorse XFe96 metabolic flux analyzer (Agilent) was used to measure OCR. Stimulated mouse B cells were plated (3x10^5^ cells/well) in Seahorse XF RPMI medium supplemented with 2mM L-glutamine, 10mM glucose and 1mM sodium pyruvate onto CellTak- (Corning) coated XFe96 cell culture microplates (Agilent). The cells were subsequently treated with 1µM oligomycin (Thermo Scientific), 1µM FCCP, and 1µM rotenone (Cayman Chemical) + antimycin A (Sigma-Aldrich). Oxygen consumption rates (OCR, pmol/min) were monitored in real time after each inhibitor injection. For monitoring oxygen consumption levels upon BCR stimulation, Ramos B cells were plated (1x10^5^ cells/well) in Seahorse XF RPMI medium supplemented with 2mM L-glutamine, 10mM glucose, 1mM sodium pyruvate and 1.5mM CaCl_2_ onto CellTak-coated microplates. The cells were injected with anti-human IgM (5µg/ml) or media followed by 1µM rotenone + antimycin A. Oxygen consumption rates were measured repeatedly every 6.5 min for 2 h after anti-human IgM injection.

### 2.10 Quantification and statistical analysis

Statistical analysis was performed using Prism 10 (GraphPad Software). *P* values of less than 0.05 were considered statistically significant. Normality was tested using Shapiro-Wilk test. Unpaired t test or Mann-Whitney test was used for unpaired comparisons of normally and non-normally distributed data, respectively. When comparing two paired data populations, paired t test or Wilcoxon matched-pairs signed rank test was used to determine statistical significance in the case of normal and non-normal distribution, respectively. When performing multiple comparisons ANOVA and Kruskal-Wallis were used for normally distributed data and not normally distributed data respectively. The number of independent repeats is indicated in the figure legend. Experiments were considered independent if they were started on separate days.

## 3 Results

### 3.1 Mitochondrial depolarization is not sufficient to induce B cell apoptosis

**Figure 1:**
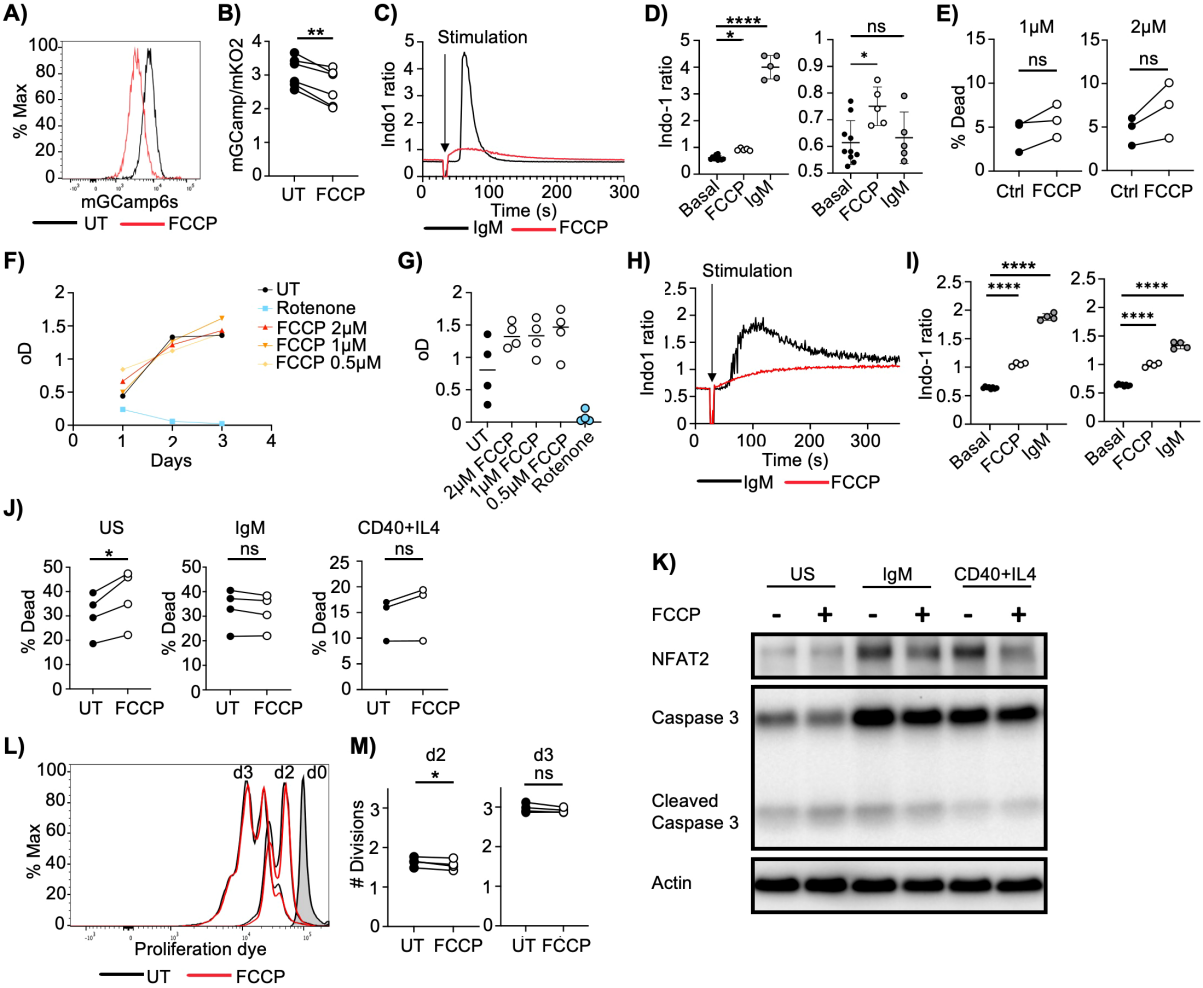
Mitochondrial depolarization does not induce apoptosis. **(A)** Mitochondrial Ca^2+^ levels in Ramos cells expressing mGCaMP6s treated with DMSO or FCCP (2µM) for 30 min. Representative of 9 measurements from 4 independent experiments. **(B)** Mitochondrial Ca^2+^ levels in Ramos B cells expressing mGCaMP6s_P2AmKO2 treated with DMSO (UT) or FCCP (2µM) for 30 min. The ratio of mGCaMP6s signal to mKO2 signal is shown as a measure of mCa^2+^ levels. (n= 6 samples from 4 independent experiments). **(C)** Cytosolic Ca^2+^ levels in Ramos cells loaded with the Ca^2+^ indicator indo-1 AM, stimulated with anti-IgM (5µg/ml) or FCCP (2µM). The ratio of fluorescence of indo-1 bound to Ca^2+^ vs free indo-1 is used as a measure for cytosolic Ca^2+^ levels. Representative of 4 independent experiments. **(D)** Summary of experiments shown in 1C. Maximal value of indo-1 ratio (left) and indo-1 ratio at 2 min (right) after FCCP and anti-IgM stimulation compared to Ca^2+^ levels before stimulation (Basal) (n= 10 Basal, 5 anti-IgM, 5 FCCP treated samples from 4 independent experiments). **(E)** Frequency of dead (zombie aqua positive) Ramos cells cultured overnight in the presence of DMSO (Ctrl) or FCCP (1 or 2µM). Pooled data from 3 independent experiments (n=3). **(F)** Cell growth measurement of Ramos cells upon FCCP treatment (0.5, 1 and 2µM) using the CCK-8 kit. Rotenone (1µM) was used as a control. Representative of 4 independent experiments. **(G)** Cell growth measurement of Ramos cells on day 2 as shown in **(F)**. Pooled data from 4 independent experiments. **(H)** Cytosolic Ca^2+^ levels as determined by Indo-1 AM staining of mouse B cells stimulated with anti-IgM (10µg/ml) or FCCP (1µM). Representative of 2 independent experiments with 4 mice in total. **(I)** Summary of experiments shown in 1H. Maximal indo-1 ratio (left) and indo-1 ratio at 4 min (right) after FCCP and anti-IgM stimulation compared to Ca^2+^ levels before stimulation (Basal) (n= 4 mice from 2 independent experiments). **(J)** Frequency of dead (zombie aqua positive) mouse B cells cultured overnight unstimulated (US) or stimulated with anti-IgM (10µg/ml) or anti- CD40 (0.2µg/ml) + IL4 (10ng/ml)) in the presence of DMSO or FCCP (1µM). (n= 4 mice from 2 independent experiments) **(K)** Analysis of the indicated protein levels. Cells were treated as in 1J (n= 4 mice from 2 independent experiments) **(L)** Measurement of mouse B cell proliferation by eFluor 670 dilution. Cells were stimulated with 10µg/ml anti- IgM in the presence of DMSO or FCCP (1µM) for 3 days. (n= 4 mice from 2 independent experiments) **(M)** Average cell division of mouse B cells shown in **(L)**. (n= 4 mice from 2 independent experiments) Data are presented as mean values. Paired Student’s t test **(B, E, J, M)** and ANOVA **(D, I)** were used for statistical analysis. *p < 0.05; **p < 0.01; ****p < 0.0001; ns, not significant.

Mitochondria are well recognized for their capacity to sequester Ca²^+^ (2). However, it remains uncertain whether B cells maintain stable mCa²^+^ levels or if these levels dynamically fluctuate in response to external stimuli. To investigate mCa²^+^ dynamics, we engineered a fluorescent, mitochondria-targeted Ca²^+^ sensor (mGCaMP6s) and introduced it into the human Burkitt lymphoma B cell line Ramos (**Supplementary Figure S1A**). This construct consists of two mitochondria targeting sequences (18, 19), a sequence encoding the green fluorescent protein (GFP) in which the amino- and carboxyl- terminal fragments are separated by a short spacer, calmodulin and calmodulin-binding peptide M13. Upon calmodulin binding of Ca^2+^ ions, two halves of GFP reassemble, producing a fluorescence signal. The Ramos B cells express an IgM-class BCR (IgM-BCR) and are used in many successful signaling studies (20–24). Upon treatment with the mitochondrial uncoupler carbonyl cyanide-p-trifluoromethoxyphenylhydrazone (FCCP), a notable decrease in the mGCaMP6s fluorescence signal was observed (**Figure 1A**), indicating the release of mCa²^+^ consequent to mitochondrial membrane potential disruption. To facilitate comparative analysis of mCa²^+^ flux across independently transduced cell lines, we developed a ratiometric version of the sensor. This construct comprises the mGCaMP6s sequence, followed by a P2A linker and a fluorescent, mitochondria-targeted, Ca²^+^-insensitive marker, mKO2 (**Supplementary Figure S1A**). Mitochondrial localization of both markers was confirmed via microscopy (**Supplementary Figure S1B**) and linked expression was verified by flow cytometry (**Supplementary Figure S1C**). Upon FCCP treatment, a reduction in mGCaMP6s fluorescence intensity was observed, while mKO2 fluorescence remained unchanged, resulting in a diminished mGCaMP6s/mKO2 ratio (**Figures 1B**, **Supplementary Figure S1D**). These experiments confirm that B cell mitochondria harbor substantial levels of Ca^2+^.

The loss of mitochondrial membrane potential is a central event in apoptosis, and mCa²^+^ release has been implicated in the activation of pro-apoptotic pathways (25–27). To investigate whether mCa²^+^ release upon mitochondrial depolarization is sufficient to induce cell death in Ramos cells, we first examined cytosolic Ca²^+^ influx following FCCP treatment. We observed that FCCP triggered a cytosolic Ca²^+^ flux that, while substantially lower in magnitude, was significantly more prolonged compared to rapid Ca²^+^ mobilization from the ER and extracellular sources induced by anti-IgM stimulation (**Figures 1C, D**). These results demonstrate that mitochondrial uncoupling induces cytosolic Ca²^+^ accumulation with kinetics distinct from BCR-mediated Ca²^+^ signaling. Subsequently, we assessed cell survival and proliferation after FCCP treatment. Despite effective mitochondrial depolarization, Ramos cells remained viable and continued to proliferate (**Figures 1E–G**). To determine if resting B cells exhibit similar resilience to mitochondrial depolarization, we isolated naïve mouse B cells and confirmed that FCCP treatment leads to mCa²^+^ release into the cytosol (**Figure 1H**). Consistent with observations in Ramos cells, cytosolic Ca²^+^ levels after FCCP treatment in mouse B cells were significantly lower than those elicited by anti-IgM stimulation (**Figure 1I**).

Next, we evaluated the impact of FCCP on survival and proliferation in naïve and activated B cells. FCCP modestly increased cell death in unstimulated B cells, but had no effect on cells activated with anti-IgM or anti-CD40 plus IL-4 (**Figure 1J**, **Supplementary Figure S1E**). Furthermore, levels of cleaved caspase 3, a key apoptosis marker, remained unchanged following FCCP treatment (**Figure 1K**, **Supplementary Figure S1F**). Similarly, proliferation of anti-IgM–stimulated B cells was unaffected by FCCP (**Figures 1L, M**). Finally, we examined whether FCCP-induced mCa²^+^ release influences the protein levels of NFAT2, a Ca²^+^-dependent transcription factor known to regulate its own expression via a positive feedback loop and to accumulate in B cells with enhanced cytosolic Ca²^+^ signaling (28, 29). Our data indicate that mCa²^+^ release alone does not elevate NFAT2 protein levels (**Figure 1K**, **Supplementary Figure S1F**).

In summary, our findings demonstrate that while FCCP treatment induces mitochondrial depolarization and mCa²^+^ release, this intervention is insufficient to activate apoptotic signaling in B cells.

### 3.2 Mitochondrial depolarization inhibits BCR-dependent signaling

**Figure 2:**
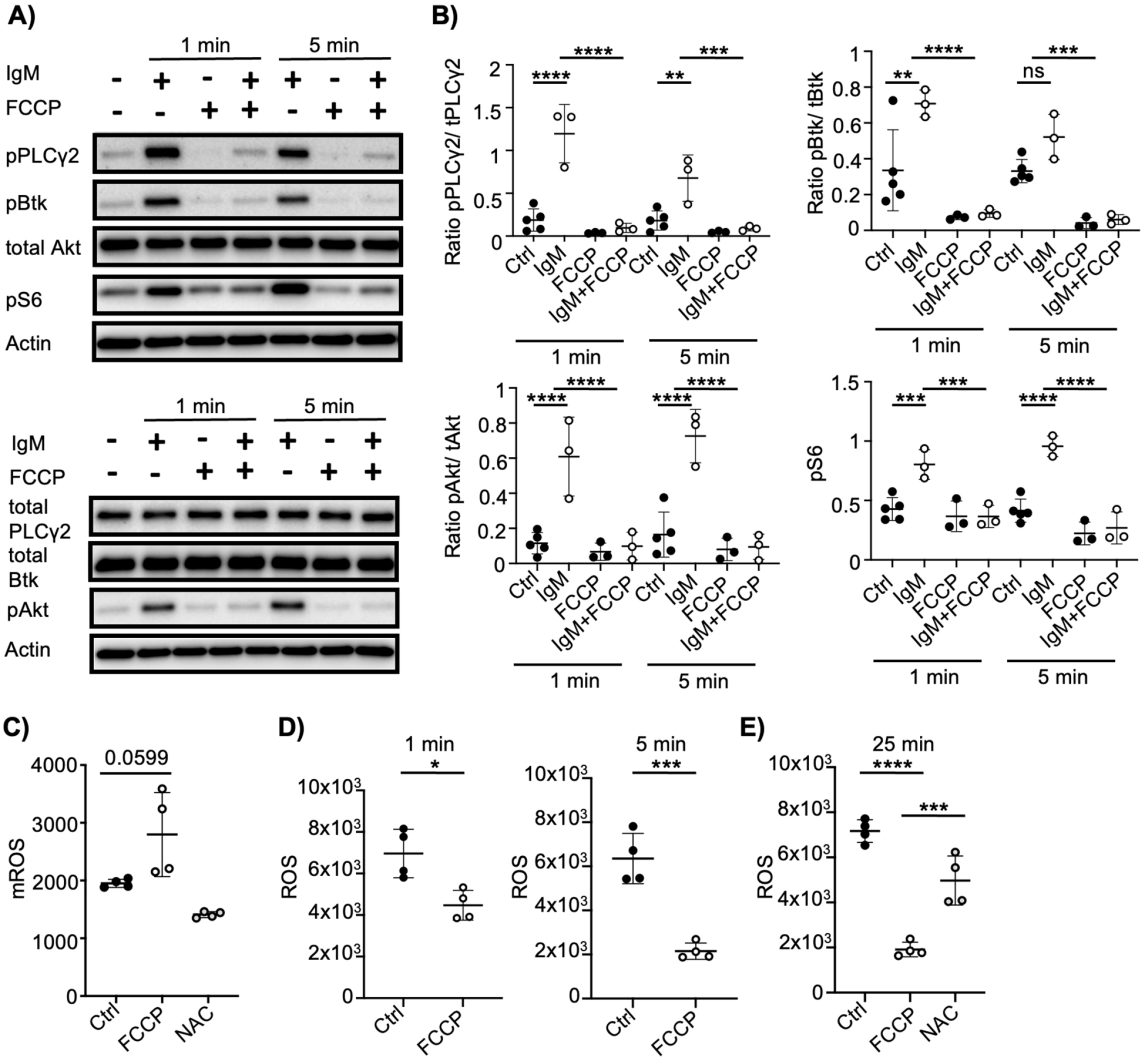
Mitochondrial depolarization inhibits BCR-dependent signaling. **(A)** Mouse B cells were stimulated with anti-IgM (10µg/ml) or FCCP (2µM) or a combination of both for the indicated time in minimal media. Shown are levels of pPLCγ2(Y1217), pBtk(Y223), pAkt(S473), pS6(S235/236), PLCγ2, Btk, Akt and actin. Representative of 3 independent experiments. For these experiments, prepared lysates were loaded on two gels which were run in parallel to assess total and phopho- protein levels. Equal loading is verified by probing for actin. **(B)** Quantification of pPLCγ2/PLCγ2, pBtk/Btk, pAkt/Akt, pS6 band intensities shown in **(A)**. Both phospho- and total signal intensities were first normalized to actin and then phospho- proteins were normalized to their respective total protein signals. Pooled data from 3 independent experiments. **(C)** Mitochondrial ROS levels of mouse B cells loaded with MitoSOX red for 10 min and simultaneously treated with DMSO, FCCP (2µM) or N-acetyl cytsteine (NAC) in minimal media. (n=4 mice from 2 independent experiments) **(D)** ROS levels of mouse B cells loaded with CellROX deep red in minimal media for 25 min and then treated with DMSO or FCCP (2µM) for indicated times. (n=4 mice from 2 independent experiments) **(E)** ROS levels of mouse B cells loaded with CellROX deep red for 25 min and simultaneously treated with DMSO, FCCP (2µM) or NAC in minimal media. (n=4 mice from 2 independent experiments) Data are presented as mean. ANOVA **(B, E)** and unpaired Student’s t test **(C, D)** were used to compare groups. *p < 0.05; **p < 0.01; ***p < 0.001; ****p < 0.0001; ns, not significant.

Cytosolic Ca²^+^ not only facilitates the activation of transcription factors such as NFAT2 but also plays a critical role in driving proximal BCR signaling. To determine whether the FCCP-induced increase in cytosolic Ca²^+^ alone activates these signaling pathways or modulates anti-IgM–induced signaling, we analyzed the activation of key signaling molecules. Unexpectedly, FCCP treatment markedly diminished the phosphorylation of various BCR-proximal signaling molecules, including PLCγ2, Btk, Akt, and S6 (**Figures 2A, B**).

Beyond mCa²^+^ release, mitochondrial depolarization can influence the generation of ROS, which are an essential amplifier of BCR signaling (6). We therefore examined ROS levels following FCCP treatment. While mitochondrial ROS levels exhibited a slight increase (**Figure 2C**), total cellular ROS levels were significantly reduced (**Figures 2D, E**) in FCCP treated mouse B cells. Similar to mouse B cells, total ROS levels also declined upon FCCP exposure in Ramos cells (**Supplementary Figures S2A, B**).

To ensure that FCCP does not directly interfere with the fluorescence of the ROS-detecting dye CellROX, we evaluated its signal in a cell-free system containing hydrogen peroxide, FCCP, or the solvent DMSO. As anticipated, hydrogen peroxide enhanced fluorescence, whereas FCCP and DMSO had no significant effect (**Supplementary Figure S2C**).

Collectively, these findings indicate that FCCP disrupts redox homeostasis in B cells, correlating with impaired BCR signaling. Notably, the impact of FCCP on BCR signaling was influenced by the culture conditions. The aforementioned experiments were conducted in minimal media; however, when cells were stimulated in complete media, the inhibitory effects on signaling were attenuated (**Supplementary Figure S2D**), and ROS levels declined more gradually (**Supplementary Figures S2E, F**). Furthermore, preincubation with FCCP followed by stimulation in complete media reinstated the reduction in signaling (**Supplementary Figures S2G, H**). Proliferation remained unaffected under these conditions, although a modest decrease in cell death was observed (**Supplementary Figures S2I, J**).

In summary, mitochondrial depolarization modulates B cell signaling, potentially through ROS reduction. Moreover, the experimental context, particularly medium composition, can modulate the functional effects of mitochondrial depolarization.

### 3.3 BCR signaling induces mitochondrial Ca^2+^ uptake

**Figure 3:**
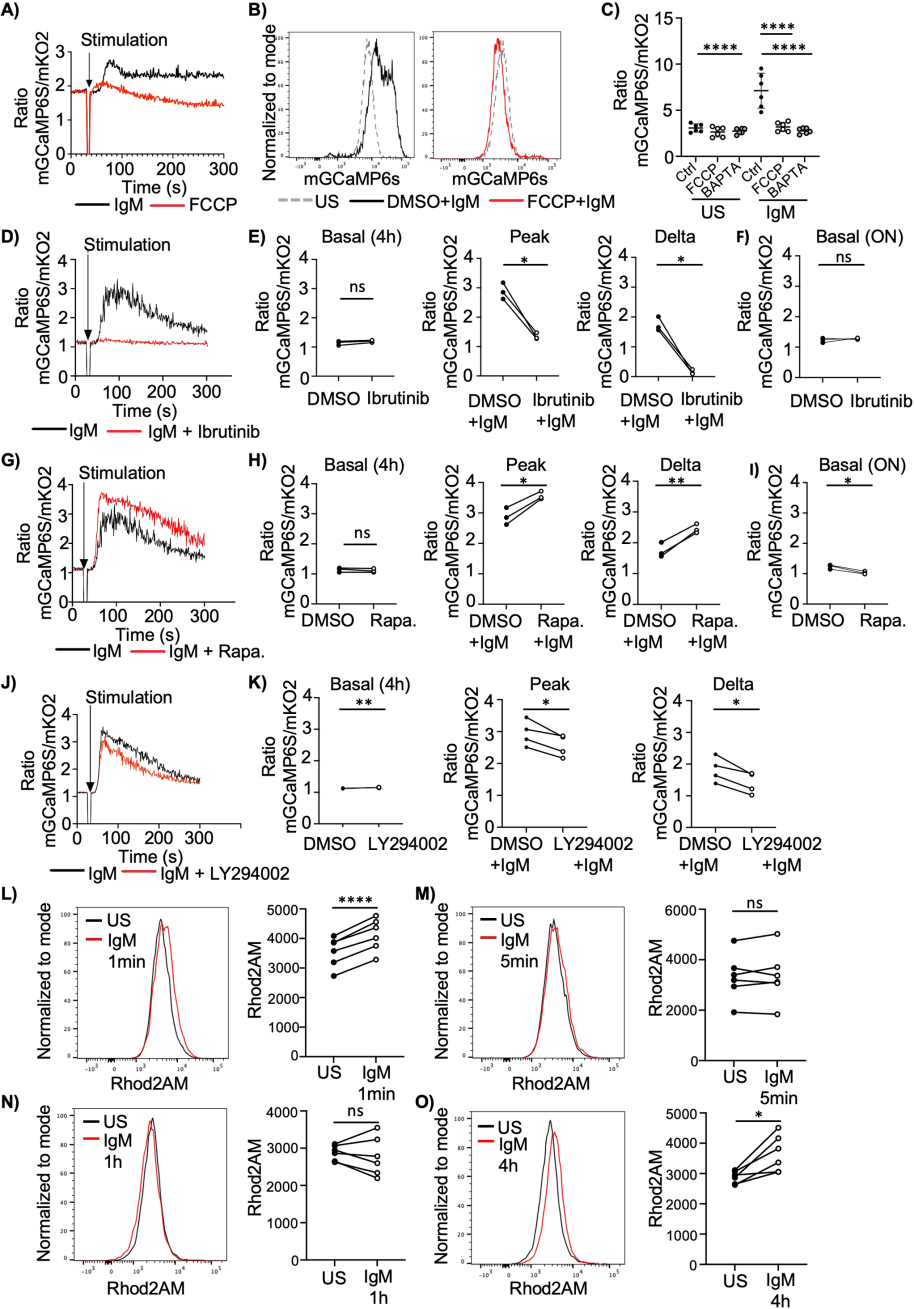
BCR signaling induces mitochondrial Ca^2+^ uptake. **(A)** Mitochondrial Ca^2+^ in Ramos cells expressing mGCaMP6s_mKO2 stimulated with anti- IgM (5µg/ml) or FCCP (2µM). Representative of 3 independent experiments. **(B)** Mitochondrial Ca^2+^ levels in Ramos cells expressing mGCaMP6s treated with DMSO or FCCP (2µM) for 30 min and stimulated with anti- IgM (5µg/ml) for 1 minute. (n=9 samples from 4 independent experiments) **(C)** Mitochondrial Ca^2+^ levels in Ramos cells expressing mGCaMP6s_mKO2 treated with DMSO, FCCP (2µM) or BAPTA-AM for 30min and stimulated with anti- IgM (5µg/ml) for 1min. The ratio of mGCaMP6s signal to mKO2 signal represents mCa^2+^ levels. (n=6 samples from 4 independent experiments) **(D, G, J)** Mitochondrial Ca^2+^ in Ramos cells expressing mGCaMP6s_mKO2 pre-treated with DMSO, Ibrutinib (2µM, D) rapamycin (100nM, G) or LY294002 (25µM,J) for 4h and stimulated with anti-IgM (5µg/ml). Representative of 3 independent experiments. **(E, H, K)** Summary showing basal, peak and the difference between basal and peak (delta) mCa^2+^ levels from experiments shown in **(D, G, J)**. **(F, I)** Mitochondrial Ca^2+^ levels in Ramos cells expressing mGCaMP6s_mKO2 treated with DMSO, Ibrutinib (2µM, F) and rapamycin (100nM, I) overnight. The ratio of mGCaMP6s signal to mKO2 signal represents mCa^2+^ levels. Data represent 3 independent experiments. **(L, M)** Left: mCa^2+^ levels in mouse B cells loaded with Rhod-2 AM for 20 min and stimulated with anti- IgM (10µg/ml) for 1 **(L)** and 5 min **(M)**. Right: Summary of Rhod-2 AM mean fluorescence intensity. (n=6 mice in 3 independent experiments) **(N, O)** Left: mCa^2+^ in mouse B cells stimulated with anti-IgM (10µg/ml) for 1 **(N)** and 4 h **(O)** and loaded with Rhod-2 AM for 20 min. Right: Summary of Rhod-2 AM mean fluorescence intensity. (n=6 mice in 3 independent experiments) Data are presented as mean. ANOVA **(C)** and paired Student’s t test **(E, F, H, I, K, L, M, N, O)** were used to compare groups. *p < 0.05; **p < 0.01; ****p < 0.0001; ns, not significant.

Upon BCR stimulation, Ca²^+^ is released from the ER to activate Ca²^+^-dependent signaling pathways (1). As discussed above, we have demonstrated that mitochondria can release Ca²^+^ without compromising B cell function. Building on this, we sought to determine whether mitochondria, like the ER, also release Ca²^+^ upon B cell activation. To this end, Ramos cells expressing the mitochondrial Ca²^+^ sensor mGCaMP6S_mKO2 were stimulated with anti-IgM. We observed an immediate increase in the mGCaMP6S/mKO2 fluorescence ratio following stimulation, indicative of mCa²^+^ uptake (**Figure 3A**). Although the initial mCa²^+^ peak rapidly declined, stimulated cells maintained elevated mitochondrial Ca²^+^ levels for a prolonged period compared to unstimulated controls (**Figures 3B, C**). Similar trends were observed using the mitochondrial Ca^2+^-sensitive dye Rhod2-AM, albeit with lower sensitivity to mCa²^+^ fluctuations (**Supplementary Figures S3A, B**).

Next, we investigated whether the BCR-induced rise in mCa²^+^ depends on activation of proximal BCR signaling pathways. Treatment with Ibrutinib, a Btk inhibitor, effectively suppressed phosphorylation of PLCγ2, Akt, and S6 (**Supplementary Figures S3C, D**) and completely abolished anti-IgM–induced mitochondrial Ca²^+^ uptake (**Figures 3D, E**), but did not affect steady state mCa^2+^ levels (**Figure 3F**). Conversely, inhibition of mTORC1 with rapamycin enhanced activation-induced mCa²^+^ uptake (**Figures 3G, H**) but decreased steady-state levels (**Figure 3I**). Lastly, we tested LY294002, an inhibitor of PI3K. We used the inhibitor at a concentration where Akt phosphorylation was strongly reduced (**Supplementary Figure S3F**) and found that mCa²^+^ uptake was significantly reduced however not as dramatically as after Btk inhibition (**Figures 3J, K**).

In addition to cell activation, the microenvironment might also affect how B cells respond to stimulation. To investigate whether the metabolic environment influences mitochondrial Ca²^+^ uptake we chose two conditions that are physiologically relevant to B cell function: taurine treatment and hypoxia. Taurine is an amino acid found in human serum and has previously been reported to affect cellular metabolism (30). Taurine serum levels are increased in various B cell driven autoimmune disorders (31, 32), however whether taurine can affect B cell function is currently unknown. We found that an overnight incubation with taurine modestly reduced basal mCa²^+^ levels (**Supplementary Figure S3G**). Interestingly, cells pretreated with taurine for 4 h exhibited attenuated mCa²^+^ responses upon anti-IgM stimulation relative to untreated controls (**Supplementary Figure S3H**). In contrast, culturing cells under hypoxic conditions resulted in elevated mitochondrial Ca²^+^ uptake (**Supplementary Figure S3I**). B cell access to oxygen can vary depending on the tissue and niche and hypoxic regions have been reported to be found in the germinal center (33–35). In summary these experiments show that the metabolic composition of the microenvironment can affect mCa^2+^ uptake.

To assess whether similar mitochondrial Ca²^+^ dynamics occur in resting B cells as in Ramos cells, we stimulated naïve mouse B cells with anti-IgM and detected a significant increase in mCa²^+^ within 1 minute, as measured by Rhod2-AM staining (**Figure 3L**). The Ca^2+^ chelator BAPTA-AM was used as a control to verify that the increase in Rhod2-AM signal is Ca^2+^ dependent (**Supplementary Figure S3J**). Notably, mCa²^+^ levels returned to baseline after 5 min before rising again in a secondary wave at later time points (**Figures 3M–O**). Human resting B cells exhibited comparable mCa²^+^ uptake kinetics to mouse B cells upon anti-IgM activation (**Supplementary Figure S3K**).

In summary, mitochondrial Ca²^+^ levels increase following BCR stimulation and are modulated by both signaling- and metabolic-cues.

### 3.4 BCR signaling induces the formation of mitochondria-ER contact sites

**Figure 4:**
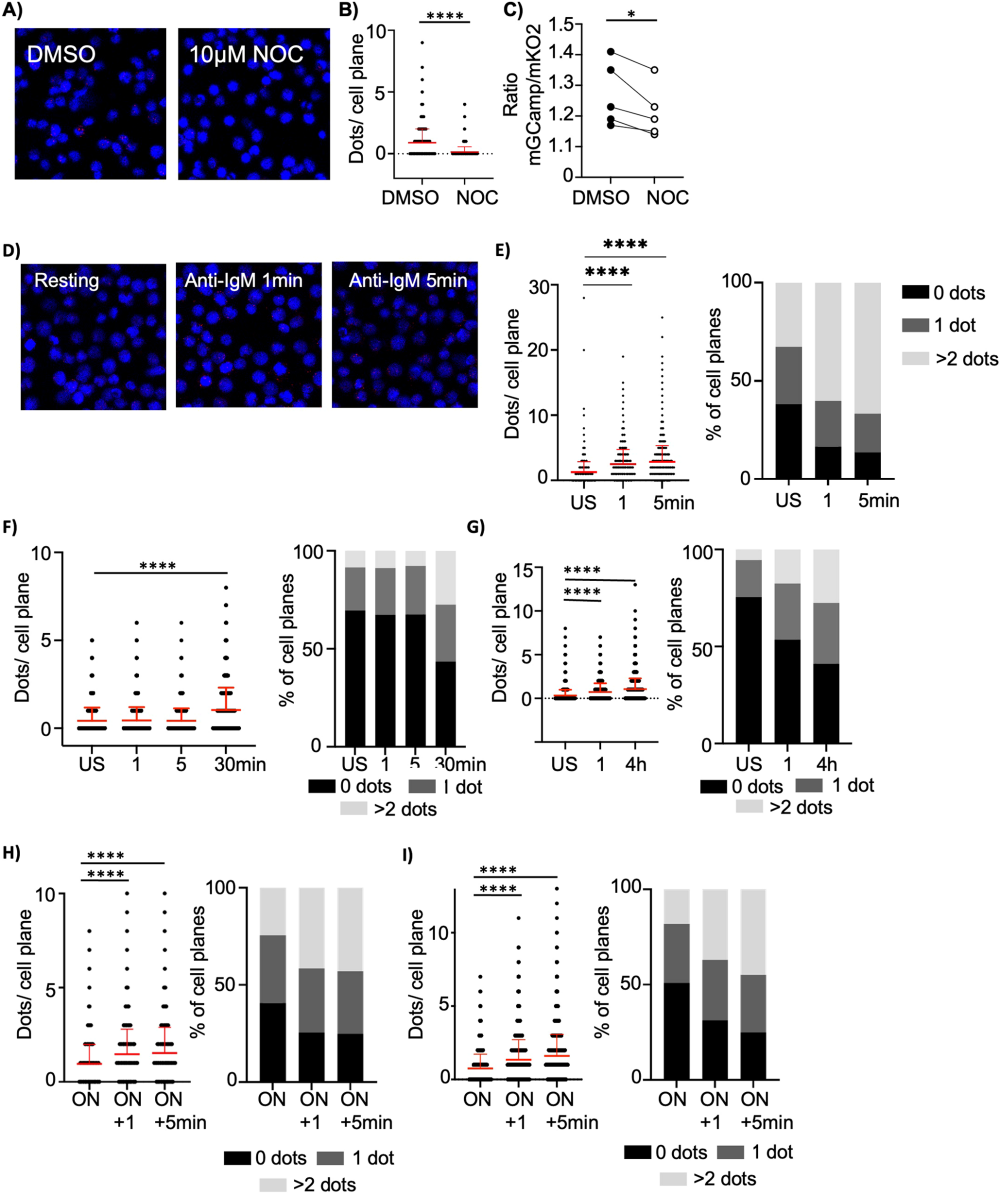
BCR signaling induces the formation of mitochondria-ER contact sites. **(A)** Representative PLA images of Ramos cells treated with DMSO or nocodazole (NOC) for 12 h. Cells were probed with α-VDAC1 and α-IP3R1 antibodies. Red dots show mitochondria-ER contact sites. Cell nuclei were counterstained with DAPI (blue). (representative for n=3 samples per condition from 2 independent experiments) **(B)** Quantification of dots per cell plane of PLAs shown in **(A)**. (n=3 from 2 independent experiments) **(C)** Mitochondrial Ca^2+^ levels of Ramos cells expressing mGCaMP6_mKO2 treated with DMSO or nocodazole for 12 h. The ratio of mitoGCaMP6s signal to mKO2 signal represents mCa^2+^ levels. (n=5 from 4 independent experiments). **(D)** Representative PLA images of Ramos cells stimulated with anti-IgM (5µg/ml) for the indicated time. Cells were treated as in **(A)**. (n=3 from 2 independent experiments). **(E)** Left: Quantification of PLA shown in **(D)**. Right: Percentage of cell planes with 0, 1 and more than 2 (>2) dots. (representative for n=3 samples per condition from 2 independent experiments) **(F)** Left: quantification of mitochondria-ER contact sites in mouse B cells stimulated with anti- IgM (10µg/ml) for the indicated time. Cells were stained as in **(A)**. Right: Percentage of cell planes with 0, 1 and more than 2 (>2) dots. (representative for n=4 mice from 2 independent experiments) **(G)** Left: quantification of mitochondria-ER contact sites in mouse B cells stimulated with anti- IgM (10µg/ml) for the indicated time. Cells were stained as in **(A)**. Right: Percentage of cell planes with 0, 1 and more than 2 (>2) dots. Representative of n=5 (for 1 h) and n=6 (for unstimulated and 4 h) from 3 independent experiments. **(H)** Left: quantification of mitochondria-ER contact sites in mouse B cells pre-stimulated with anti-CD40 (5µg/ml) + IL4 (10ng/ml) overnight and subsequently stimulated with anti- IgM (10µg/ml) for the indicated time. Cells were stained as in **(A)**. Right: Percentage of cell planes with 0, 1 and more than 2 (>2) dots. Representative of n=4 from 2 independent experiments. **(I)** Left: quantification of mitochondria-ER contact sites of mouse B cells pre-stimulated with LPS (5µg/ml) overnight and stimulated with anti- IgM (10µg/ml) for the indicated time. Cells were stained as in **(A)**. Right: Percentage of cell planes with 0, 1 and more than 2 (>2) dots. Representative of n=4 from 2 independent experiments. Data are presented as mean. Mann-Whitney U **(B, F)** and paired Student’s t test **(C)** and Kruskal-Wallis test **(E, G, H, I)** were used to compare groups. *p < 0.05; ****p < 0.0001.

In various cell types, it has been demonstrated that the ER and mitochondria establish specialized, highly organized contact sites (36). These sites facilitate inter-organelle communication and can be visualized using proximity ligation assays (PLA) targeting the mitochondrial channel VDAC and the ER-resident receptor IP3R. Performing PLA on Ramos cells, we identified pre-existing ER-mitochondria contact sites, which were disrupted upon treatment with nocodazole (**Figures 4A, B**). Nocodazole is known to interfere with the cytoskeletal structures that help maintain ER-mitochondria interaction (37). Nocodazole treatment not only diminished the number of contact sites but also reduced mCa²^+^ levels, implicating that the junctions may play a role in mCa²^+^ uptake (**Figures 4A–C**). Alternatively, however, nocodazole also affects other aspects of Ca^2+^ homeostasis (38) and could affect mCa^2+^ levels via junction-independent mechanisms as well.

We next explored whether IgM-BCR stimulation modulates the formation of these contact sites. Remarkably, within one minute of anti-IgM stimulation, the number of contact sites in Ramos cells significantly increased (**Figures 4D, E**, **Supplementary Figure S4A**). As with steady-state mCa^2+^ levels, nocodazole pre-treatment largely prevented BCR-stimulation induced mCa^2+^ uptake (**Supplementary Figures S4B, C**) as well as cytosolic Ca^2+^ accumulation (**Supplementary Figures S4D, E**). Similarly to Ramos cells, naïve mouse B cells exhibited an increase in contact site numbers upon anti-IgM stimulation, though with slower kinetics. A notable rise was observed only after 30 min, followed by a progressive increase over time (**Figures 4F, G**, **Supplementary Figures S4F, G, H**).

Given the more rapid response in Ramos cells compared to resting mouse B cells, we hypothesized that cellular pre-activation accelerates contact site formation upon BCR engagement. Indeed, overnight exposure of murine B cells to the co-stimulatory signals anti-CD40 and IL-4 primed the cells, resulting in a swift increase in contact sites within one minute of subsequent anti-IgM stimulation (**Figure 4H**, **Supplementary Figure S4I**). A similar effect was observed with LPS preactivation (**Figures 4I**, **Supplementary Figure S4J**).

In summary, our findings reveal that BCR activation promotes the dynamic formation of ER-mitochondria contact sites, potentially facilitating mitochondrial Ca²^+^ uptake during B cell activation.

### 3.5 The mitochondrial calcium uniporter driven Ca^2+^ import shapes mitochondrial responses

**Figure 5:**
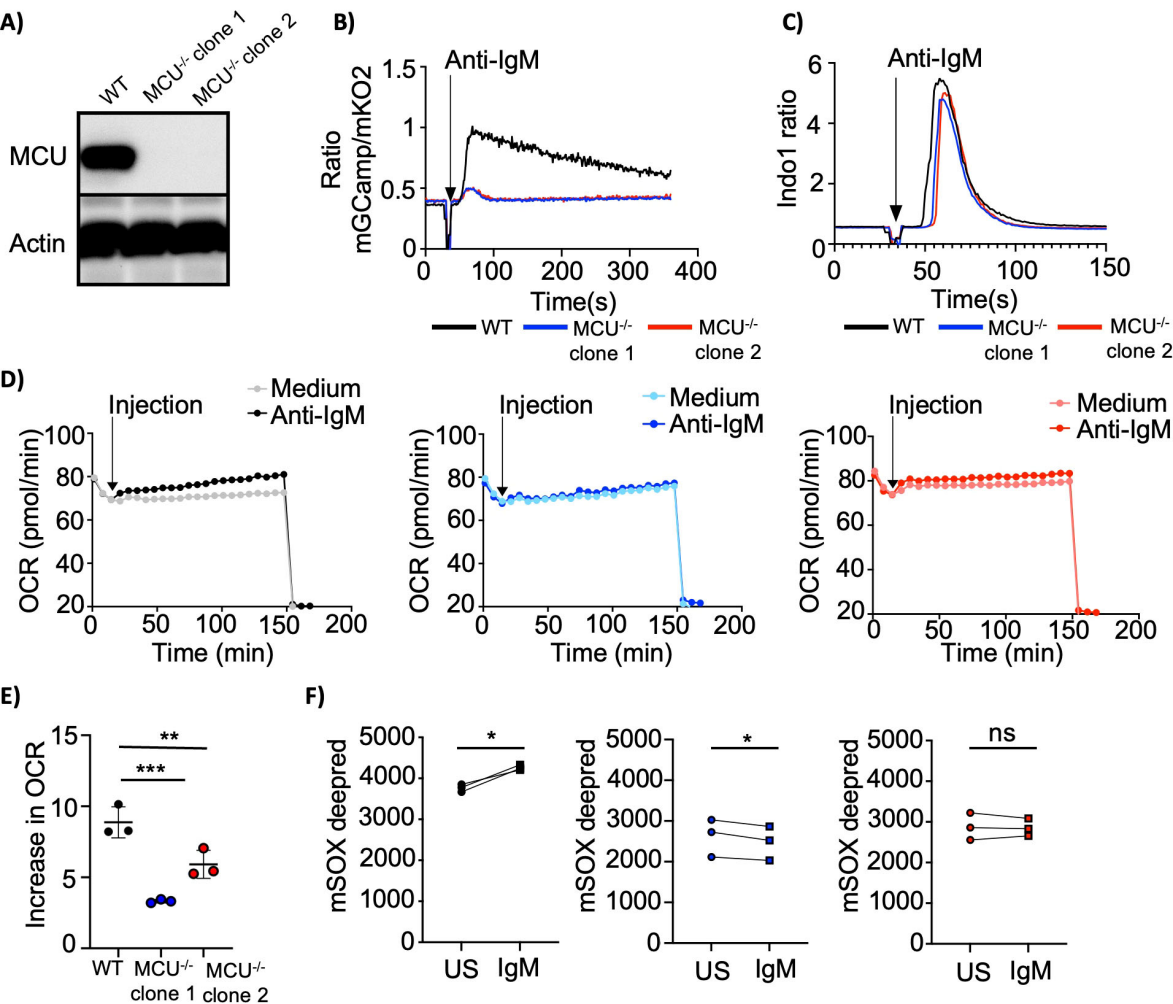
MCU regulates mitochondrial activity and cell signaling in anti-IgM stimulated B cells. **(A)** Representative immunoblot analysis of MCU protein levels in wild-type Ramos cells and two MCU-deficient cell clones. Actin was used as loading control. Representative of 3 independent experiments. **(B)** Mitochondrial Ca^2+^ in wild-type and MCU-deficient Ramos cells expressing mGCaMP6s_mKO2 stimulated with anti-IgM (5µg/ml). Representative of 6 independent experiments. **(C)** Cytosolic Ca^2+^ levels in the cells as in **(B)** loaded with indo-1 AM for 30 min and stimulated with anti- IgM (5µg/ml). Representative of 6 independent experiments. **(D)** Oxygen consumption rates (OCR) in the cells as described in **(B)**. Cells were stimulated with anti- IgM (5µg/ml) or media after 15min of measurement, followed by rotenone + antimycin A injection after 150min of measurement. OCR was measured using Seahorse flux technology. The measurement was performed in technical quadruplicates (anti-IgM injection) and triplicates (media injection). Representative of 3 independent experiments. **(E)** Summary of OCR from experiments as shown in **(D)**. Shown is the increase in OCR after anti-IgM stimulation compared to media addition. To calculate this value the basal OCR was first subtracted from the maximal OCR and then the value obtained from the media control was subtracted from the value obtained from the anti-IgM stimulated cells. Pooled data from 3 independent experiments. **(F)** Mitochondrial ROS levels of cells as described in **(B)**. Cells were loaded with mtSOX deep red for 30 min and stimulated with anti- IgM (5µg/ml) for 1 min. Pooled data from 3 independent experiments. Data are presented as mean. ANOVA **(E)** and paired Student’s t test **(F)** were used to compare groups. *p < 0.05; **p < 0.01; ***p < 0.001; ns, not significant.

The mitochondrial calcium uniporter (MCU) is the central subunit of the MCU complex, which is widely regarded as the primary transporter responsible for importing Ca²^+^ into the mitochondrial matrix. To determine whether BCR-induced mCa²^+^ uptake depends on MCU, we generated Ramos cells with a targeted deletion of the MCU gene (**Figure 5A**) and found that BCR induced mCa^2+^ uptake was largely dependent on MCU (**Figure 5B**). In contrast, cytosolic Ca²^+^ mobilization was only slightly reduced by MCU deletion (**Figure 5C**, **Supplementary Figure S5A**).

Within mitochondria, MCU operates as part of a multiprotein complex including MCUR1 that functions as a regulator of MCU activity (39). To assess whether MCUR1 influences mCa²^+^ uptake in B cells, we deleted MCUR1 in Ramos cells (**Supplementary Figure S5B**) and found that BCR-induced mCa²^+^ uptake occurred largely independently of MCUR1 (**Supplementary Figures S5C, D**).

Mitochondria harbor numerous Ca²^+^-dependent enzymes, notably within TCA cycle and potentially the electron transport chain (2). To investigate whether mitochondrial oxygen consumption relies on MCU-mediated Ca²^+^ uptake, we measured the oxygen consumption rate (OCR) using a Seahorse metabolic analyzer. Directly after anti-IgM stimulation, wild-type Ramos cells exhibited a modest increase in OCR, whereas MCU-deficient cells displayed a trend toward a less pronounced response (**Figures 5D, E**). Given that mitochondria are a major source of ROS and that MCU has been implicated in maintaining redox balance in other contexts, we examined ROS levels in MCU-deficient Ramos cells. MCU^-/-^ Ramos cells showed reduced basal ROS production (**Supplementary Figure S5E**) and, in contrast to wild-type cells, failed to upregulate ROS generation following anti-IgM stimulation (**Figure 5F**).

In summary, while the loss of MCU-mediated mCa²^+^ uptake after BCR activation only marginally impacts oxygen consumption, it profoundly diminishes ROS production, underscoring a critical role for MCU in redox regulation.

### 3.6 Cytosolic Ca^2+^ increase is sufficient to induce mCa^2+^ uptake

**Figure 6:**
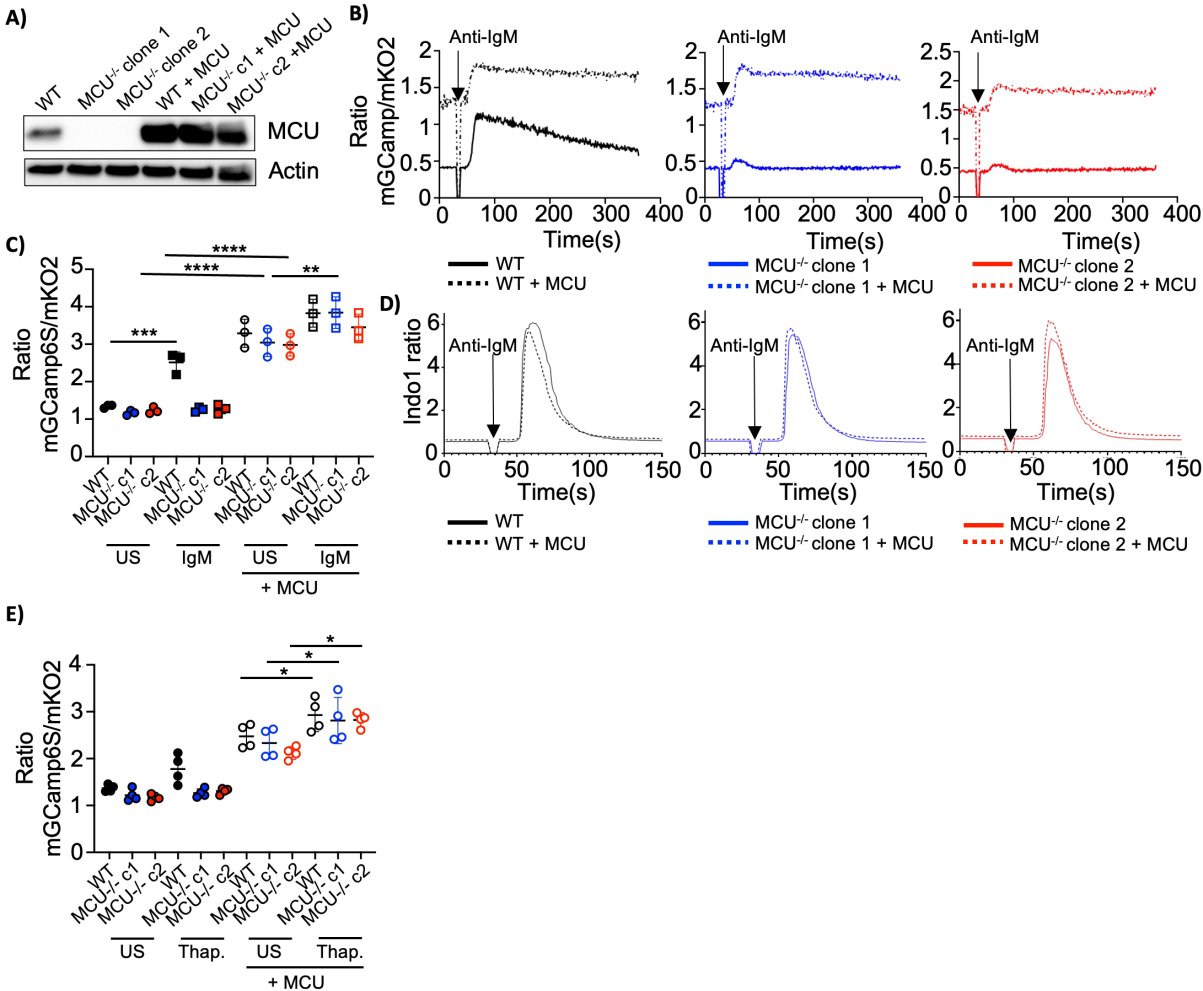
MCU protein levels and cytosolic Ca^2+^ dictate mCa^2+^ uptake. **(A)** Representative immunoblot of MCU protein levels in wild-type, MCU-deficient and MCU overexpressing Ramos cells. Actin is used as a loading control. Representative of 2 independent experiments. **(B)** Mitochondrial Ca^2+^ in wild-type, MCU-deficient and MCU-overexpressing Ramos cells expressing mGCaMP6s_mKO2 stimulated with anti- IgM (5µg/ml). Representative of 4 independent experiments. **(C)** Summary of mCa^2+^ levels in cells as described in **(B)** stimulated in HBSS with anti-IgM (5µg/ml) for 1 min. Pooled data from 3 independent experiments. **(D)** Cytosolic Ca^2+^ levels in cells as described in **(B)** loaded with indo-1 AM and stimulated with anti- IgM (5µg/ml). Representative of 4 independent experiments. **(E)** Mitochondrial Ca^2+^ levels in cells as described in **(B)** treated with DMSO or thapsigargin for 1 min. Pooled data from 4 independent experiments. Data are presented as mean. ANOVA **(C, E)** was used to compare groups. *p < 0.05; **p < 0.01; ***p < 0.001; ****p < 0.0001.

Mitochondrial Ca^2+^ uptake via MCU has been reported to be repressed under steady-state conditions by the Ca^2+^ binding MCU regulators MICU1 and MICU2 (40). High Ca^2+^ concentrations disinhibit MCU channels allowing mCa^2+^ uptake (40). To test whether mCa^2+^ uptake is limited by MCU protein expression or by cytosolic Ca^2+^ levels in B cells we first overexpressed MCU in Ramos cells (**Figure 6A**). MCU overexpressing cells displayed an increase in mCa^2+^ levels at steady-state but were able to further increase mCa^2+^ uptake upon BCR stimulation (**Figures 6B, C**). In contrast, cytosolic Ca^2+^ was not substantially affected by the overexpression of MCU (**Figures 6D**, **Supplementary Figure S6A**). Of note, MCU overexpression restored the ability to increase ROS production in MCU-deficient clones (**Supplementary Figure S6B**). Mitochondrial Ca^2+^ has been suggested to play a role in cell death induction (25), however we did not observe significant changes in apoptosis or proliferation in MCU-deficient or overexpressing cells (**Supplementary Figures S6C, D**). Despite changes in ROS production upon anti-IgM stimulation, we did not observe strong changes in BCR proximal signaling in MCU-deficient or MCU overexpressing cells (**Supplementary Figures S6E, F**). MCU has been reported to drive mitochondrial biogenesis (41), but we did not observe any significant differences in mitochondrial or ER mass (**Supplementary Figures S6G, H**). As previously demonstrated, BCR-activation induces mCa^2+^ uptake with a kinetic similar to cytosolic Ca^2+^ mobilization. We next tested whether cytosolic Ca^2+^ accumulation in response to BCR-independent stimuli is sufficient to induce mCa^2+^ uptake or whether activation of signaling molecules downstream of the BCR are needed. To this end, we treated the cells with thapsigargin, an inhibitor of the ER Ca^2+^ pump, which causes ER stress and cytosolic Ca^2+^ accumulation. Upon thapsigargin treatment, wild-type Ramos cells promptly accumulated Ca^2+^ within their mitochondria, whereas MCU-deficient Ramos cells were unable to do so (**Figure 6E**). Cells overexpressing MCU exhibited elevated basal mCa^2+^ levels and retained the capacity to further augment mCa^2+^ uptake upon treatment (**Figure 6E**).

In summary, our findings indicate that mitochondrial Ca^2+^ levels are regulated not only by the abundance of MCU protein but also by cytosolic Ca^2+^ concentrations.

### 3.7 MCU regulates mitochondrial activity and cell signaling in anti-IgM stimulated B cells

We have established that MCU is essential for mCa²^+^ uptake and shapes mitochondrial function in Ramos B cells. To determine whether MCU plays a comparable role in non-transformed B cells, we crossed *Mcu^fl/fl^* mice with a B cell-specific, tamoxifen-inducible Cre-expressing mouse line (*Mb1^CreERT2^*) (42). To delete MCU in B cells, we employed two distinct experimental setups.

**Figure 7:**
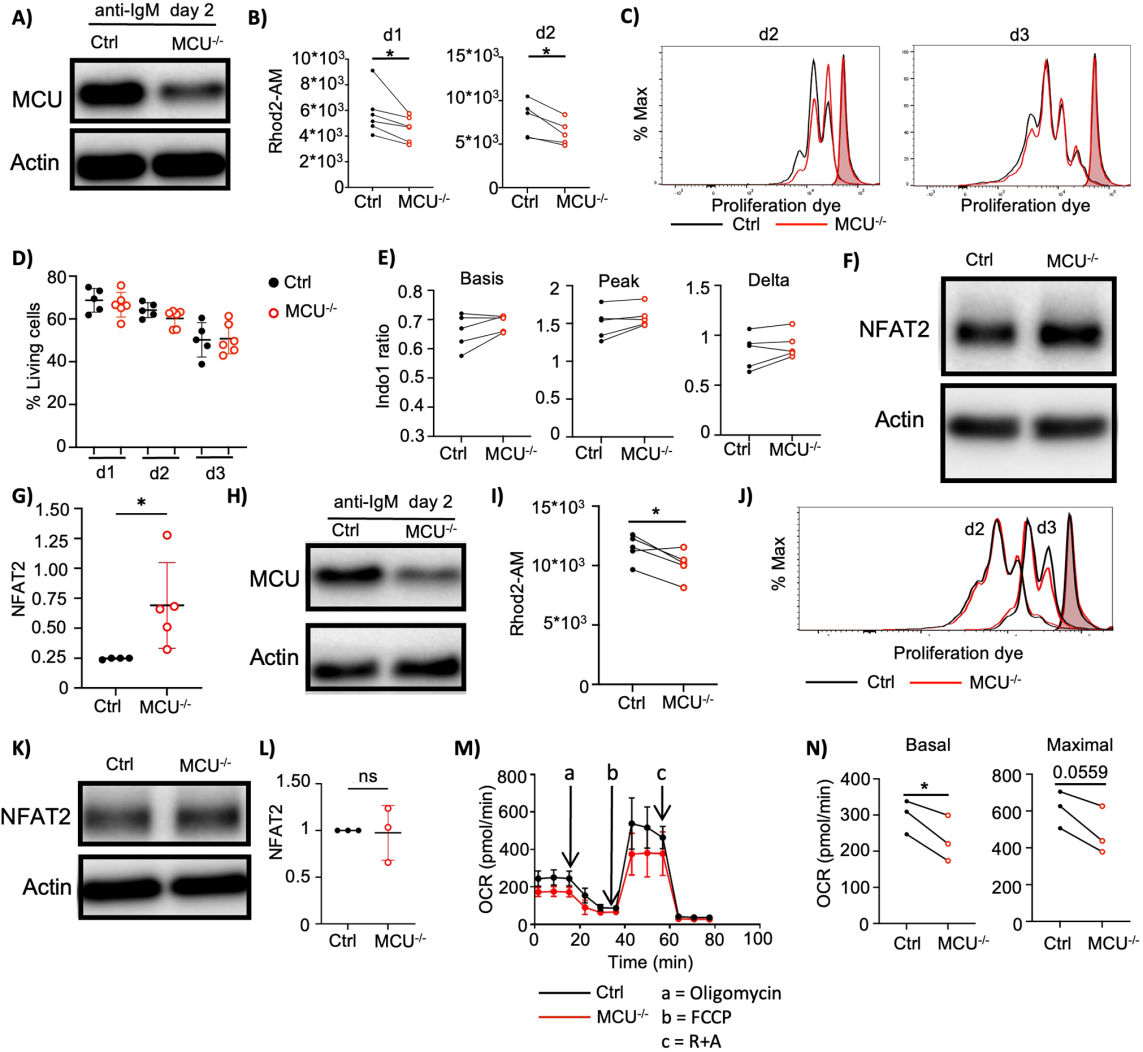
The loss of MCU alters cell signaling and oxygen consumption. **(A–G)***Mcu^fl/fl^ x mb1^CreERT2^* (MCU^-/-^) and control (Ctrl) mice were injected with tamoxifen for three consecutive days to induce B cell-specific *Mcu* deletion. **(A)** Representative immunoblot analysis of MCU protein levels in MCU^-/-^ and Ctrl mouse B cells stimulated with anti-IgM for 2 days. Representative of 3 independent experiments. **(B)** Mitochondrial Ca^2+^ levels in MCU^-/-^ and Ctrl mouse B cells stimulated with anti-mouse IgM (10µg/ml) for 1 (left) and 2 days (right) were determined by Rhod-2 AM staining. Pooled data from 6 and 5 independent experiments, respectively. (d1: n=9 for MCU^-/-^ and 10 for Ctrl, d2: n=8 for MCU^-/-^ and Ctrl mice). **(C)** Measurement of mouse B cell proliferation by eFluor 670 dilution. MCU^-/-^ and Ctrl mouse B cells were stimulated with anti- IgM and tracked for 3 days. Representative of 3 independent experiments. (n=6 MCU^-/-^ and 5 Ctrl mice). **(D)** Cell survival analysis of mouse B cells shown in **(C)**. Forward scatter (FSC) and side scatter (SSC) properties were used to determine the percentage of living cells. Pooled data from 3 experiments. (n=6 MCU^-/-^ and 5 Ctrl mice). **(E)** Cytosolic Ca^2+^ was assessed in MCU^-/-^ and Ctrl B cells using indo-1 AM. Shown are basal and peak Ca^2+^ levels after anti-IgM stimulation as well as the difference between peak and basal levels (delta). Pooled data from 5 independent experiments. (n=7 for MCU^-/-^ and for Ctrl). **(F)** Representative immunoblot analysis of NFAT2 protein levels in MCU^-/-^ mouse B cells compared to Ctrl B cells. Cells were stimulated with anti-mouse IgM (10µg/ml) for 48 h. Blots were probed for NFAT2 and actin. Representative of 3 independent experiments. **(G)** Quantification of **(F)** was performed by normalization of NFAT2 levels to actin levels. Then, fold change in NFAT2 levels of MCU^-/-^ mouse B cells relative to Ctrl B cells was calculated. Pooled data from 3 independent experiments. (n=5 Ctrl and 7 MCU^-/-^ mice). **(H–N)***Mcu^fl/fl^* and Ctrl B cells were treated with TAT-CRE and stimulated with anti-IgM. **(H)** Representative immunoblot analysis of MCU protein levels in MCU^-/-^ and Ctrl mouse B cells stimulated with anti-IgM for 48h. Blots were probed with MCU and actin antibodies. Representative of 3 independent experiments. **(I)** Mitochondrial Ca^2+^ levels in MCU^-/-^ and Ctrl mouse B cells stimulated as in **(H)** was determined with Rhod-2 AM staining. Pooled data from 5 independent experiments. (n=6 MCU^-/-^ and 7 Ctrl mice). **(J)** MCU^-/-^ and Ctrl B cells were stimulated with anti-IgM. Proliferation was assessed by eFluor 670 dilution. Representative of 3 independent experiments. **(K)** Representative immunoblot analysis of NFAT2 protein expression in MCU^-/-^ and Ctrl mouse B cells shown in **(H)**. Blots were probed for NFAT2 and actin. Representative of 3 independent experiments. **(L)** Quantification of **(K)** was performed by normalization of NFAT2 levels to actin levels. Then, fold change of NFAT2 levels of MCU-deficient mouse B cells relative to Ctrl B cells was calculated. Pooled data from 3 independent repeats. (n=4 for MCU^-/-^ and Ctrl mice).**(M)** Representative oxygen consumption measurement of MCU^-/-^ and Ctrl mouse B cells as in **(H)**. Oxygen consumption rates (OCR) were measured by Seahorse flux technology after sequential injection of oligomycin (1µM), FCCP (1µM) and rotenone + antimycin A (1µM). Representative of 3 independent experiments. **(N)** Summary graphs showing calculated basal and maximal respiration in anti-IgM stimulated MCU-deficient and Ctrl mouse B cells shown in **(M)**. Pooled data from 3 independent experiments. (n=3 for MCU^-/-^ and Ctrl). Data are presented as mean. Paired **(B, I, N)** and unpaired **(G, L)** Student’s t tests were used to compare groups. *p < 0.05; ns, not significant.

In the first approach, mice received tamoxifen injections on three consecutive days and were sacrificed shortly thereafter. B cells were then purified and stimulated *in vitro* with anti-IgM. Tamoxifen treatment reduced MCU protein levels in *Mcu^fl/fl^ x mb1^CreERT2^*B cells, however the deletion was not complete (**Figure 7A**, **Supplementary Figures S7A, B**). Using the mCa²^+^ indicator Rhod2AM, we confirmed that MCU is required for mCa²^+^ uptake, as anti-IgM-stimulated MCU-deficient B cells exhibited significantly lower mCa²^+^ levels compared to controls (**Figure 7B**). We did not observe significant changes to mCa^2+^ levels in B cells purified from tamoxifen treated cells at steady state levels (**Supplementary Figure S7C**). Ca²^+^ homeostasis and mitochondrial integrity have previously been implicated in regulating apoptosis upon anti-IgM stimulation (17). Anti-IgM stimulation has been shown to result in cytosolic Ca^2+^ overload and mitochondrial bloating as evidenced by an increase in Mitotracker Green staining (17). Thus, we sought to test whether reduced mCa²^+^ levels in anti-IgM stimulated MCU deficient cells affect mitochondrial mass, cell survival and proliferation. We observed only a slight, non-significant decrease in mitochondrial mass on day one post-stimulation and no differences after two days (**Supplementary Figure S7D**). Despite markedly reduced mCa²^+^ levels, neither cell survival nor proliferation were affected (**Figures 7C, D**, **Supplementary Figures S7E, F**), nor were there significant changes in oxygen consumption rates (**Supplementary Figures S7G, H**). We next assessed whether MCU deletion impacts cytosolic Ca^2+^ signaling. Immediately following anti-IgM stimulation, MCU-deficient B cells showed a modest trend toward increased cytosolic Ca^2+^ mobilization (**Figure 7E**), but no differences were evident after one or two days (**Supplementary Figure S7I**). Similarly, ER mass remained unchanged (**Supplementary Figure S7J**). We observed a marked increase in NFAT2 levels (**Figures 7F, G**), a Ca²^+^-responsive transcription factor that accumulates in response to heightened cytosolic Ca^2+^ signaling, indicating that MCU deletion has the potential to modulate cytosolic signal transduction.

Previous studies suggest that lymphocytes may adapt to the genetic loss of MCU, potentially masking certain phenotypes over time (43). To address whether prolonged deletion obscures phenotypic effects, we implemented a second, more acute experimental setup. Here, purified *Mcu^fl/fl^* B cells were exposed to TAT-CRE, a cell permeable Cre enzyme. Directly after TAT-CRE treatment, the cells were stimulated with anti-IgM. In this setup MCU protein is still present during anti-IgM stimulation and the effects of MCU loss in the subsequent phases of stimulation is investigated. In this setting, *Mcu* gene deletion efficiency and MCU protein levels were reduced to a similar degree as observed with the first setup (**Figure 7H**, **Supplementary Figures S7K, L**) and mCa²^+^ levels were significantly reduced on day 2 of stimulation (**Figure 7I**). Consistent with the first setup, we observed no differences in proliferation (**Figure 7J**, **Supplementary Figure S7M**), mitochondrial mass (**Supplementary Figure S7N**), ER mass (**Supplementary Figure S7O**) or cytosolic Ca²^+^ levels (**Supplementary Figures S7P, R**). Unlike in the first approach, there were no alterations in cytosolic NFAT2 accumulation (**Figures 7K, L**), suggesting that absence of MCU during the initial phase of BCR activation is necessary for induction of NFAT2 accumulation. Notably, we found oxygen consumption to be significantly reduced in MCU deficient cells in this setup (**Figures 7M, N**). In summary, MCU regulates mCa²^+^ uptake in non-transformed B cells as well as in transformed B cells. While B cells in general exhibit plasticity to adapt to MCU loss, MCU has the capacity to modulate mitochondrial function and cellular signaling pathways.

## 4 Discussion

It is well established that mitochondria store substantial amounts of Ca^2+^ (2). Changes in mCa²^+^ levels have long been studied primarily in the context of pathological conditions. Several pro-apoptotic treatments have been found to trigger cell death through Ca^2+^ dependent mechanisms such as Ca^2+^ induced opening of the permeability transition pore (25). Opening of this pore allows Ca^2+^ and other matrix components to exit the mitochondria. In the cytosol, a variety of Ca^2+^ activated effector molecules can participate in apoptosis induction (25). Thus, mCa^2+^ overload as well as subsequent release into the cytosol can trigger cell death. In B cells, stimulation via the BCR has been shown to result in a gradual accumulation of intracellular Ca^2+^ ultimately inducing mitochondrial dysfunction and cell death, which can be prevented by providing the cells with co-stimulatory signals (17). In addition to its pro-apoptotic role, mCa^2+^ has also been reported to boost mitochondrial activity, by stimulating Ca^2+^ dependent metabolic enzymes in the TCA cycle as well as the electron transport chain (2). We have shown before that treatment with the Ca^2+^ chelator BAPTA-AM acutely inhibits mitochondrial oxygen consumption in B cells, but not medium acidification (22). In the present study we further explored the role of mCa^2+^ dynamics in B cell activation, metabolism, signaling and functional regulation. We show that mitochondrial depolarization and subsequent increase in cytosolic Ca^2+^ neither induces activation of Ca^2+^ dependent signaling pathways, nor does it trigger apoptosis in cells cultured in complete media. In contrast, FCCP treatment in minimal media results in inhibition of signaling pathways downstream of the BCR, possibly via redox dysregulation. We found mitochondrial ROS to be increased upon FCCP treatment. In contrast, we observed a reduction of cytosolic ROS, which could be a compensatory response to an increase in mitochondrial ROS. Cytosolic ROS is crucial for BCR signaling (6), thus reduced cytosolic ROS levels are consistent with decreased BCR signaling. Complete media contains various nutrients that can shape redox balance. We observed a slower reduction in ROS levels following FCCP treatment in complete media in comparison to minimal media, which was accompanied by a delayed inhibition of signaling. These findings are significant as they demonstrate that mitochondrial depolarization and the release of mCa²^+^ alone are insufficient to trigger Ca^2+^ dependent pro-apoptotic signaling pathways. Furthermore, mitochondrial depolarization may influence B cell responses through ROS, in a manner that is shaped by the surrounding microenvironment. The contribution of mitochondria to B cell responses may be underestimated, as *in vitro* experiments are typically conducted under nutrient-rich and redox-balanced conditions that do not fully reflect the physiological environment.

We demonstrate that BCR activation induces mCa^2+^ uptake in two distinct phases. The initial phase occurs rapidly and coincides with the rise in cytosolic Ca^2+^ levels, while the second phase proceeds more slowly, with Ca^2+^ accumulating progressively over time. Both phases of mCa^2+^ uptake rely on MCU, and overexpression of MCU alone is sufficient to elevate steady-state mCa^2+^ levels. Unlike in other cell types (39), mCa^2+^ uptake is not dependent on the regulatory subunit MCUR1 in B cells. In addition to MCU protein levels, the abundance of ER-mitochondria contact sites shapes mCa^2+^ responses. Upon BCR stimulation, the number of contact sites per cell increases, likely to support the second wave of mCa^2+^ uptake. Notably, the formation of contact sites in preactivated B cells occurs rapidly within 1 min in contrast to resting B cells where this process is much slower. We have observed that nocodazole treatment, which depolymerizes microtubules, not only reduces ER–mitochondria contact sites but also diminishes mCa^2+^levels in resting and anti-IgM stimulated Ramos cells. However, since nocodazole also affects cytosolic Ca^2+^ mobilization (38), it is possible that mCa^2+^ levels are affected by nocodazole treatment independently of its effect on junction formation.

The uptake of Ca²^+^ by mitochondria upon activation may serve to enhance mitochondrial function and boost ATP production, since several of the TCA cycle enzymes are Ca^2+^ dependent (2). Indeed, deletion of MCU results in slightly reduced oxygen consumption upon BCR stimulation and significantly reduced ROS production in Ramos cells. Despite changes in ROS production, BCR proximal signaling was not affected by MCU-deletion or MCU overexpression. Several scenarios are possible to explain the lack of signaling defects upon reduced ROS production. ROS are produced by various enzymes including membrane bound NADPH oxidases and the mitochondria (6). While we observed a decrease in ROS, subcellular ROS compartmentalization might in underappreciated by these experiments. It is possible that ROS production proximal to the BCR was not affected by MCU-deletion and enabled normal activation of BCR dependent signaling. In future research, it might be valuable to analyze redox-sensitive proteins proximal to the mitochondria rather than the BCR. Alternatively, using western blots as a technique to study BCR signaling might not allow to observe subtle differences in the duration and kinetics of signaling molecule activation. We observed a subtle reduction in the peak of cytosolic Ca^2+^ mobilization in MCU-deficient Ramos cells upon BCR stimulation, which is consistent with reduced ROS production. Thus, the observed defect in ROS production might have only subtle effects on BCR signaling.

In mouse B cells, we observed no changes in oxygen consumption if MCU was deleted a few days before the experiment, but significantly reduced mitochondrial activity, if oxygen consumption was measured early after MCU deletion. Combined, these data suggest that if MCU driven Ca^2+^ uptake is abrogated, B cells learn to adapt over time. Compensatory adaptation to reduced mCa^2+^ uptake could also explain that proliferation was not affected by the loss of MCU in any condition investigated. Our findings are consistent with previously published studies showing that a chronic deletion of MCU early during lymphocyte development does not dramatically alter cell function (5, 43). Nonetheless, emerging evidence indicates that the relatively mild functional phenotype of lymphocytes might be a consequence of adaptive mechanisms that can compensate for the loss of MCU over time. For example, in contrast to a chronic deletion, acute downregulation of MCU has been found to impair suppressive capacity of regulatory T cells (43). The compensatory mechanisms could include upregulation of substrate supply to the mitochondria, post-translational modifications or expression changes in metabolic enzymes to preserve flux or a shift to increased reliance on complex II of the ETC which is believed to be Ca^2+^ independent (44, 45). However, it should be noted that in both experimental conditions, MCU protein remained detectable in B cells from experimental animals, although at reduced levels. Our genotyping experiments suggest that incomplete *Mcu* gene deletion appears to underlie the incomplete loss of MCU protein (**Supplementary Figures S7A, K**). In the tamoxifen inducible model, deletion efficiency could be affected by transitional B cells entering the spleen after tamoxifen exposure or insufficient tamoxifen penetration of the spleen. Additionally, chromatin accessibility of the *Mcu* gene locus might be suboptimal in resting B cells. Overall, the remaining MCU expressing cells might obscure more subtle functional differences in our experiments. It is therefore possible that our study underestimates the role of MCU in B cell function. Nevertheless, our findings demonstrate that MCU-dependent mCa^2+^ uptake has the capacity to regulate mitochondrial activity as both activated mouse B cells and Ramos cells displayed defects in mitochondrial function upon loss of MCU.

Notably, neither MCU deletion nor overexpression affected B cell survival, despite the resulting mCa^2+^ depletion or overload. Together with our observation that FCCP treatment does not compromise cell viability, these findings suggest that Ca^2+^ dysregulation alone is insufficient to initiate apoptosis in B cells. This implies that an additional signal may be required to trigger cell death in this context. Thus, dynamic mitochondrial Ca^2+^ uptake and release can occur without compromising cell viability, enabling mitochondrial Ca^2+^ to participate in the regulation of other cellular processes. We found NFAT2 protein levels to increase over time in stimulated MCU-deficient B cells. This is consistent with previous studies reporting enhanced NFAT activity following MCU deletion in lymphocytes (5). NFAT2 is a Ca^2+^ dependent transcription factor, known to drive its own expression in an autoregulatory feed-forward loop (28). An accumulation of NFAT2 protein levels could thus be a consequence of increased cytosolic Ca^2+^ signaling, which would be consistent with previously published results (5). Although we did not observe a significant difference in cytosolic Ca^2+^ mobilization upon anti-IgM stimulation, it is possible that subtle changes in Ca^2+^ dynamics are sufficient to drive NFAT2 accumulation. Moreover, when MCU deletion was induced immediately prior to B cell stimulation, NFAT2 accumulation was not observed, unlike in experiments where MCU had been deleted several days earlier. If the gene encoding MCU is deleted directly before stimulation, MCU protein is still present at the time of BCR activation and declines gradually during the culture period. Normal NFAT2 levels in this setting indicate that signaling events occurring directly after initial BCR engagement are influenced by the presence of MCU, and MCU plays a less important role in NFAT2 regulation at later stages of stimulation. Taken together, these findings suggest that functional outcomes are shaped not only by absolute Ca^2+^ levels but also by the temporal dynamics of the Ca^2+^ signal.

Our results show that BCR-engagement induces an increase in mCa^2+^ levels, which might benefit activated B cells by boosting mitochondrial function. In addition to activation, we found other cues to regulate mCa^2+^ uptake. We could show that ER stress induced by thapsigargin is sufficient to increase mCa^2+^ in an MCU-dependent manner. Since protein refolding requires ATP (46), boosting mitochondrial activity by increasing mCa^2+^ levels could help to alleviate ER stress. In addition, we found inhibition of mTORC1 via rapamycin to reduce mCa^2+^ at steady state, but surprisingly to result in increased mCa^2+^ uptake after BCR activation. MTORC1 has the capacity to affect Ca^2+^ homeostasis via multiple mechanisms including a destabilization of ER-mitochondria contact sites, autophagy induction or dysregulation of cytosolic Ca^2+^ homeostasis (47, 48). Since mTORC1 integrates both signaling and metabolic cues, its involvement in regulating mCa^2+^ levels may allow B cells to adapt to different environmental situations. Here we provide evidence that the metabolic microenvironment shapes mCa^2+^ levels. We found hypoxia to increase mCa^2+^ levels, which could be a consequence of hypoxia-induced ER-stress (49). Moreover, we found the amino acid taurine to reduce mCa^2+^ levels following BCR stimulation. Similar to mTORC1, taurine can shape mCa^2+^ levels via multiple mechanisms and has been reported to both increase and decrease mCa^2+^ levels in other cell types (50–52). B cells can be exposed to hypoxia in normal (33–35) as well as diseased tissues (53) and taurine is present in the blood with elevated levels observed in patients with different autoimmune disorders (31, 32). Thus, the investigated conditions represent metabolic cues relevant to B cell function *in vivo*.

In summary, our study shows mCa^2+^ levels to be dynamically regulated by diverse cues such as cellular signaling, metabolic conditions, and ER stress thereby allowing B cells to fine-tune mitochondrial responses. Thus, the communication between the ER and mitochondria and the modulation of mCa^2+^ levels are important regulatory mechanisms of mitochondrial function in B cells.

## Data Availability

The raw data supporting the conclusions of this article will be made available by the authors, without undue reservation.
