# Supplementary material for: Mitochondrial calcium shapes B cell signaling and mitochondrial function
Source: Front Immunol. 2025 Dec 11;16:1710128. doi: 10.3389/fimmu.2025.1710128 (PMC12738928; doi:10.3389/fimmu.2025.1710128)
Supplement: Supplementary file 1 [file DataSheet1.pdf]

Fig.S1.:

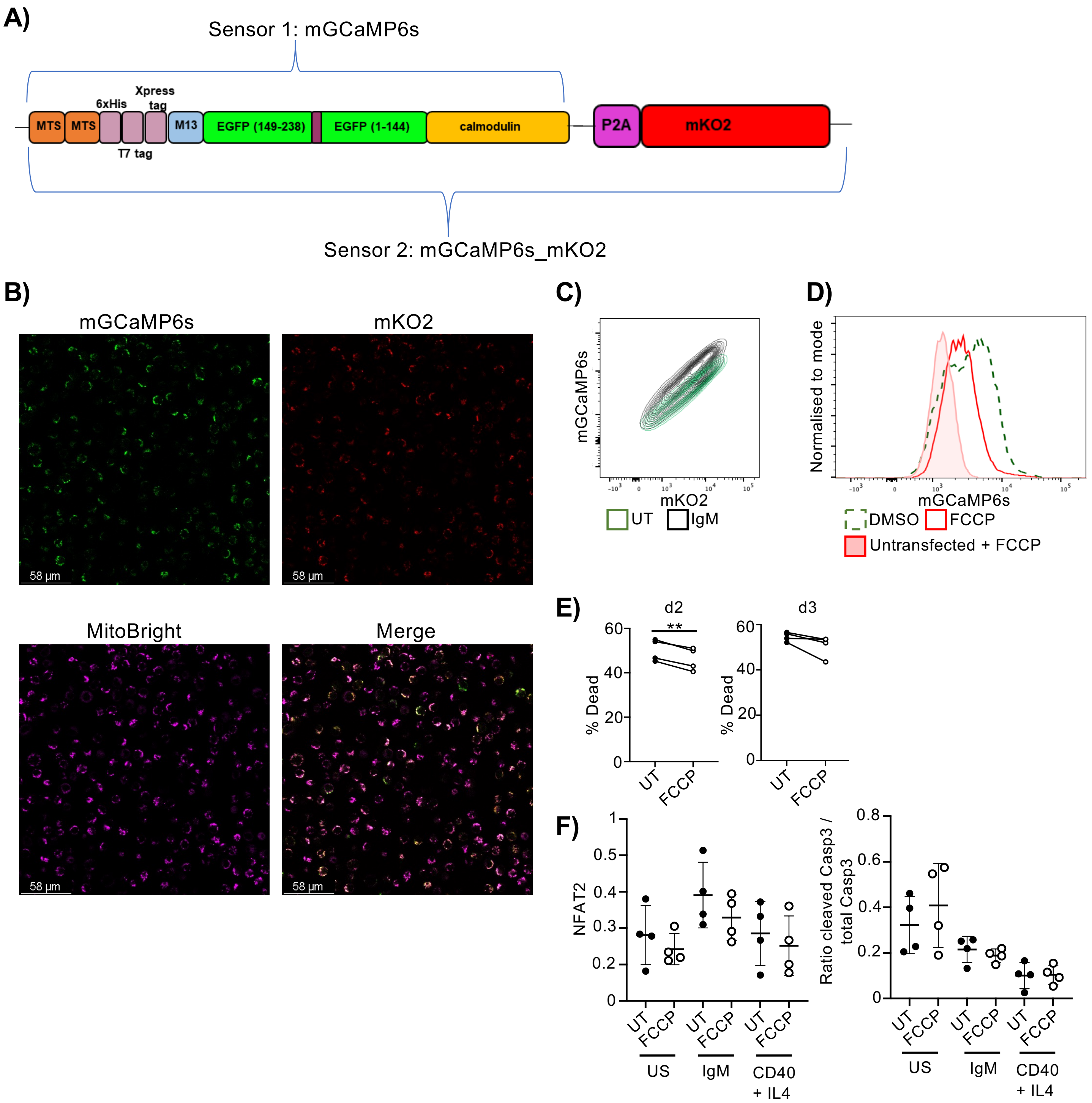


**Figure S1: Mitochondrial depolarisation does not change NFAT2 levels**

(A) Schematic representation of the mGCaMP6s and mGCaMP6s_mKO2 construct. The $mCa^{2+}$ sensor 1 (mGCaMP6s) consists of two mitochondrial targeting sequences (MTS), a green fluorescent protein (GFP) with its amino and carboxyl groups separated and reconnected by a short spacer, calmodulin and calmodulin-binding peptide, M13. Upon binding of $Ca^{2+}$ ions, two halves of GFP meet, resulting in a fluorescence signal. The $mCa^{2+}$ sensor 2 (mGCaMP6s_mKO2) is a ratiometric sensor with a viral cleavage peptide, P2A, and a $Ca^{2+}$-independent red-fluorescent protein, mKO2. The ratio between mGCaMP6s and mKO2 is used to assess $mCa^{2+}$ levels. (B) Confocal microscopy images of Ramos cells expressing mGCaMP6s_mKO2 stained with the mitochondrial mass dye MitoBright LT deep red. Representative of 3 independent experiments. (C) mGCaMP6s and mKO2 signals in Ramos cells expressing mGCaMP6s_mKO2 are shown. Shown are unstimulated and anti-IgM stimulated cells. (D) Mitochondrial $Ca^{2+}$ levels in Ramos B cells expressing mGCaMP6s_P2AmKO2 treated with DMSO or FCCP (2µM) for 30 min. For comparison non-transduced cells treated with FCCP are shown. (E) Frequency of zombie aqua-positive mouse B cells stimulated with anti-IgM in the presence of DMSO or FCCP (2uM) for 2 (left) and 3 (right) days. (n=4 mice in 2 independent experiments) (F) Summary of experiments shown in Fig.1K. Shown are NFAT2 protein levels and the ratio between cleaved caspase 3 and total caspase 3. The plot shows protein expression relative to actin. (n=4 mice from 2 independent experiments) Data are presented as mean. Paired Student's t test (E) was used to compare groups.

Fig.S2.:

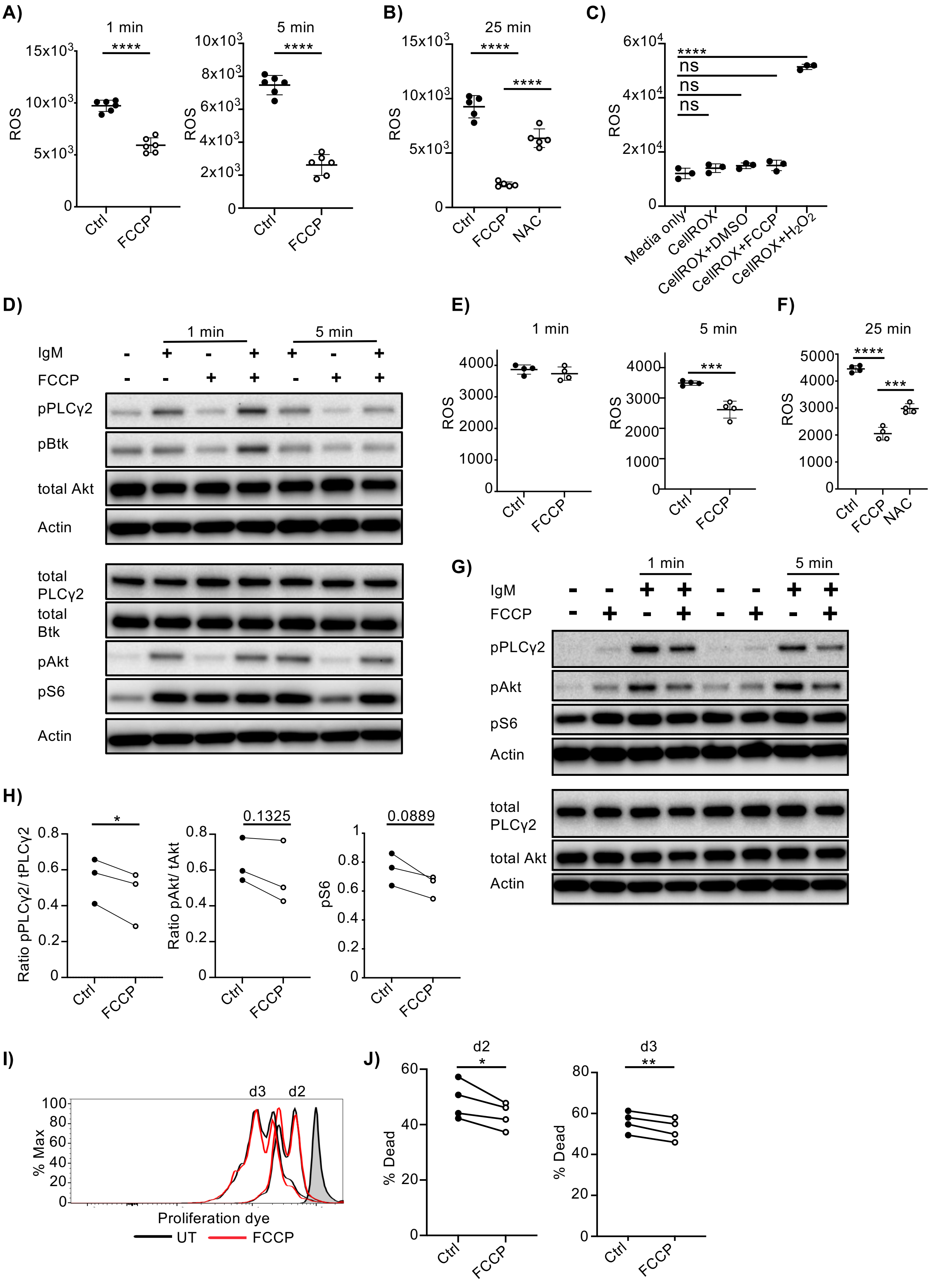

A)
1 min
5 min
ROS
Ctrl
FCCP
****
B)
25 min
NAC
C)
Media only
CellROX
CellROX+DMSO
CellROX+FCCP
CellROX+H2O2
ns
D)
IgM
FCCP
pPLCγ2
pBtk
total Akt
Actin
total PLCγ2
total Btk
pAkt
pS6
E)
***
F)
G)
H)
Ratio pPLCγ2/ tPLCγ2
Ratio pAkt/ tAkt
pS6
*
0.1325
0.0889
I)
% Max
Proliferation dye
d3
d2
UT
J)
% Dead
**

**Figure S2: The effect of mitochondrial depolarisation on BCR signaling depends on the environment**
(A) ROS levels in Ramos cells loaded with CellROX deep red in minimal media for 25 min and treated with DMSO or FCCP (2µM) for the indicated time. (n=6 samples from 3 independent experiments) (B) ROS levels of Ramos cells loaded with CellROX deep red for 25 minutes and simultaneously treated with DMSO, FCCP (2µM) or NAC in minimal media. (n=5 samples from 3 independent experiments) (C) Fluorescence intensity measurement of CellROX deep red in cell-free complete media after 5 min incubation with DMSO, FCCP (final conc. 2µM) or $H_2O_2$ (0.24%). (n=3 samples from 2 independent experiments) (D) Immunoblots of total cell lysates from mouse B cells stimulated with anti- IgM (10µg/ml) or FCCP (2µM) or a combination of both for the indicated time in complete media. Blots were probed for pPLCγ2(Y1217), pBtk(Y223), pAkt(S473), pS6(S235/236), PLCγ2, Btk, Akt and actin. Representative of 2 independent experiments. For these experiments, prepared lysates were loaded on two gels which were run in parallel to assess total and phopho-protein levels. Equal loading is verified by probing for actin. (E) ROS levels of mouse B cells loaded with CellROX deep red in complete media for 25 min and treated with DMSO or FCCP (2µM) for the indicated time. (n=4 mice from 2 independent experiments) (F) ROS levels of mouse B cells loaded with CellROX deep red for 25 min and simultaneously treated with DMSO, FCCP (2µM) or NAC in complete media. (n=4 mice from 2 independent experiments) (G) Immunoblots of total cell lysates from mouse B cells pre-treated with DMSO or FCCP (2µM) for 5 minutes and then stimulated with anti- IgM (10µg/ml) for the indicated time in complete media. Blots were probed for pPLCγ2(Y1217), pAkt(Y223), pS6(S235/236), PLCγ2, Akt and actin. Representative of 3 independent experiments. For these experiments, prepared lysates were loaded on two gels which were run in parallel to assess total and phopho- protein levels. Equal loading is verified by probing for actin (H) Quantification of immunoblot data of pPLCγ2/PLCγ2, pAkt/Akt, pS6 band intensities 5 min after anti-IgM stimulation shown in Fig.S2G. Both phospho- and total- signal intensities were first normalized to actin and then phospho- proteins were normalized to their respective total protein signals. Pooled data from 3 independent experiments. (I) Measurement of mouse B cell proliferation by eFluor 670 dilution. B cells were loaded with eFluor 670, pre-treated with DMSO or FCCP (2µM) for 5 minutes and then stimulated with 10µg/ml anti-IgM for 3 days. (n= 4 mice from 2 independent experiments) (J) Frequency of zombie aqua positive mouse B cells pre-treated with DMSO or FCCP as in Fig.S2I and stimulated with 10µg/ml anti- IgM for 2 and 3 days. (n= 4 mice from 2 independent experiments) Data are presented as mean. ANOVA (B, C, F), unpaired (A, E) and paired (H, J) Student's t tests were used to compare groups. *=p < 0.05, **=p < 0.01, ***=p < 0.001, ****=p < 0.0001, ns=not significant.

Fig.S3.:

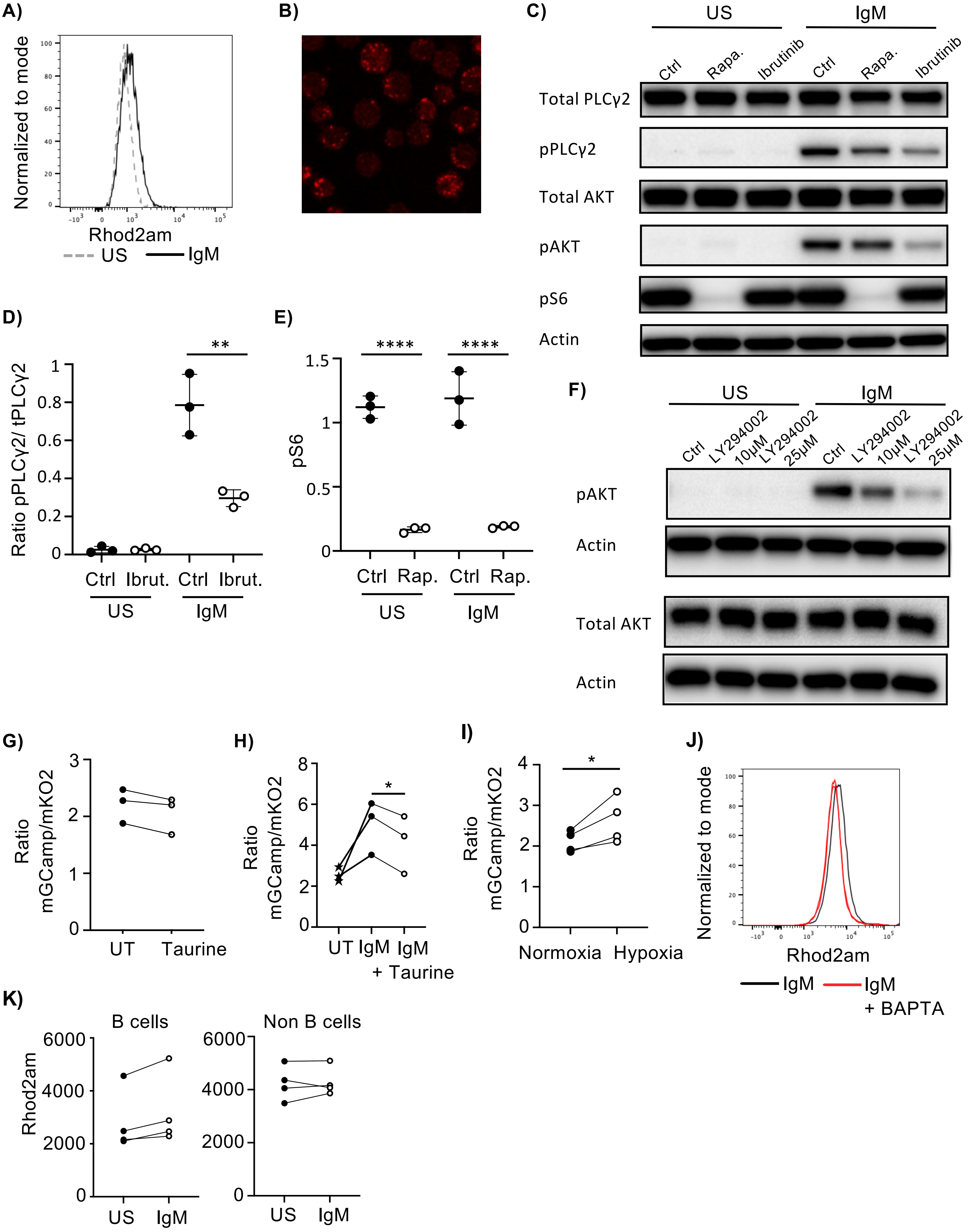
A)
Normalized to mode
Rhod2am
US
IgM
B)
C)
US
IgM
Ctrl
Rapa.
Ibrutinib
Total PLCγ2
pPLCγ2
Total AKT
pAKT
pS6
Actin
D)
Ratio pPLCγ2/ tPLCγ2
**
Ctrl Ibrut. Ctrl Ibrut.
US IgM
E)
pS6
****
****
Ctrl Rap. Ctrl Rap.
US IgM
F)
US
IgM
Ctrl
LY294002 10μM
LY294002 25μM
pAKT
Actin
Total AKT
Actin
G)
Ratio mGCamp/mKO2
UT Taurine
H)
Ratio mGCamp/mKO2
*
UT IgM IgM + Taurine
I)
Ratio mGCamp/mKO2
*
Normoxia Hypoxia
J)
Normalized to mode
Rhod2am
IgM
IgM + BAPTA
K)
B cells
Non B cells
Rhod2am
US IgM
US IgM

**Figure S3: The metabolic microenvironment shapes m$Ca^{2+}$ levels**

(A) Mitochondrial $Ca^{2+}$ levels in Ramos cells loaded with the m$Ca^{2+}$ sensitive dye Rhod-2 AM for 20 min and stimulated with anti- IgM (5µg/ml) for 1 min. Representative of 2 independent experiments. (B) Confocal microscopy images of Ramos cells loaded with Rhod-2 AM for 20 min. (n=3 in 2 independent experiments) (C) Immunoblots of total cell lysates from Ramos cells pre-treated with DMSO, rapamycin (100nM) or Ibrutinib (2µM) for 4 h and stimulated with anti- IgM (5µg/ml) for 1 min at 37 °C. Blots were probed for pPLCγ2(Y1217), pAkt(S473), pS6(S235/236), PLCγ2, Akt and actin. Representative of 3 independent experiments. Membranes were stripped after detection of phosphorylated targets and reprobed for total protein. (D, E) Quantification of immunoblot data of pPLCγ2/PLCγ2 (D) and pS6 (E) band intensities shown in Fig.S3C. Both phospho- and total signal intensities were first normalized to actin. pPLCγ2 were normalized to total PLCγ2 signal. Data represent 3 independent experiments. (F) Immunoblots of total cell lysates from Ramos cells pre-treated with DMSO or LY294002 (10 or 25µM) for 4 h and stimulated with anti- IgM (5µg/ml) for 1 min at 37 °C. Blots were probed for pAkt(S473), Akt and actin. Representative of 2 independent experiments. For these experiments, prepared lysates were loaded on two gels which were run in parallel to assess total and phopho- protein levels. Equal loading is verified by probing for actin. (G) Mitochondrial $Ca^{2+}$ levels of Ramos cells expressing mGCaMP6s_mKO2 treated with taurine (2.5µM) for 24 hours. Pooled data from 3 independent experiments. (H) Mitochondrial $Ca^{2+}$ levels of Ramos cells expressing mGCaMP6s_mKO2 cultured with taurine (2.5µM) or without taurine for 4 h and stimulated with anti- IgM for 5 min. Cells cultured without taurine and without stimulation are shown for comparison (UT) Pooled data from 3 independent experiments.(I) Mitochondrial $Ca^{2+}$ levels in Ramos cells with expressing mGCaMP6s_mKO2 cultured in either 21% (normoxia) or 4.5% (hypoxia) oxygen for 24 h. The ratio of mitoGCaMP6s signal to mKO2 signal represents m$Ca^{2+}$ levels. Pooled data from 4 independent experiments. (J) Mitochondrial $Ca^{2+}$ levels in mouse B cells stimulated with anti-IgM (10µg/ml) for 4h and loaded with Rhod-2AM while simultaneously being treated with BAPTA-AM (10µM) for 20min. Representative of 3 independent experiments. (K) Mitochondrial $Ca^{2+}$ level in human PBMCs loaded with Rhod-2 AM for 20 min and stimulated with anti- IgM (5µg/ml) for 1 min. Mitochondrial $Ca^{2+}$ levels in B cells (CD19+) and non-B cells (CD19-) were determined. Pooled data from 4 independent experiments. Data are shown as mean. ANOVA (E), unpaired (D) and paired (G, H, I) Student's t-test were used to compare groups. *= $p < 0.05$, **= $p < 0.01$, ****= $p < 0.0001$

Fig.S4.:

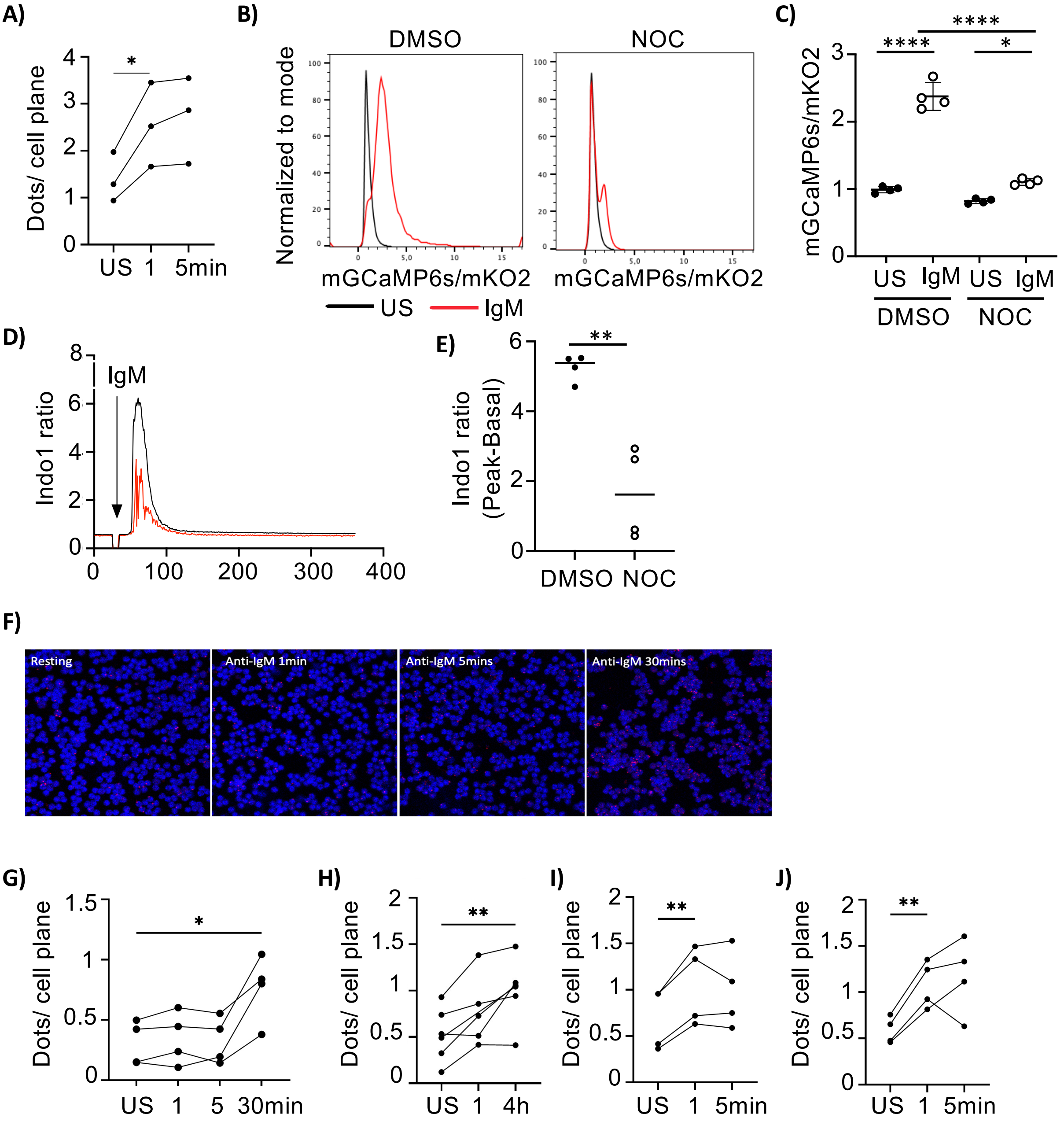


**Figure S4: BCR signaling induces the formation of mitochondria-ER contact sites.**
(A) Time course analysis of PLA experiments shown in Fig. 4E. (n=3 from 2 independent experiments). B-E) Ramos B cells expressing mGCaMP6s_mKO2 were pretreated with nocodazole for 12h and stimulated with anti-IgM. B+C) Shown is the ratio of mGCaMP6s to mKO2 after 1min. of anti-IgM stimulation. A representative experiment is shown in B), the summarised data are shown in C) (n=4 from 2 independent experiments). D+E) Shown is the Indo1 ratio as a measure of cytosolic $Ca^{2+}$ levels. An example experiment is shown in D), the summary of the experiments is shown in E). Results are shown as the difference between the value obtained at the peak of the response and the basal value (n=4 from 2 independent experiments). (F) Representative images of PLA experiments shown in Fig. 4F (n=4 from 2 independent experiments) (G) Time course analysis of PLA experiments shown in Fig. 4F. (n=4 from 2 independent experiments) (H) Time course analysis of PLA experiments shown in Fig. 4G. Pooled data from 3 independent experiments (n=5 for 1 h, n=6 for unstimulated and 4 h). (I, J) Time course analysis of PLA experiments shown in Fig. 4H and 4I. (n=4 from 2 independent experiments) Data are presented as mean. ANOVA (C), unpaired (E) and paired Student's t test (A, G-J) were used to compare groups. *= $p < 0.05$, **= $p < 0.01$, ****= $p < 0.0001$.

Fig.S5.:

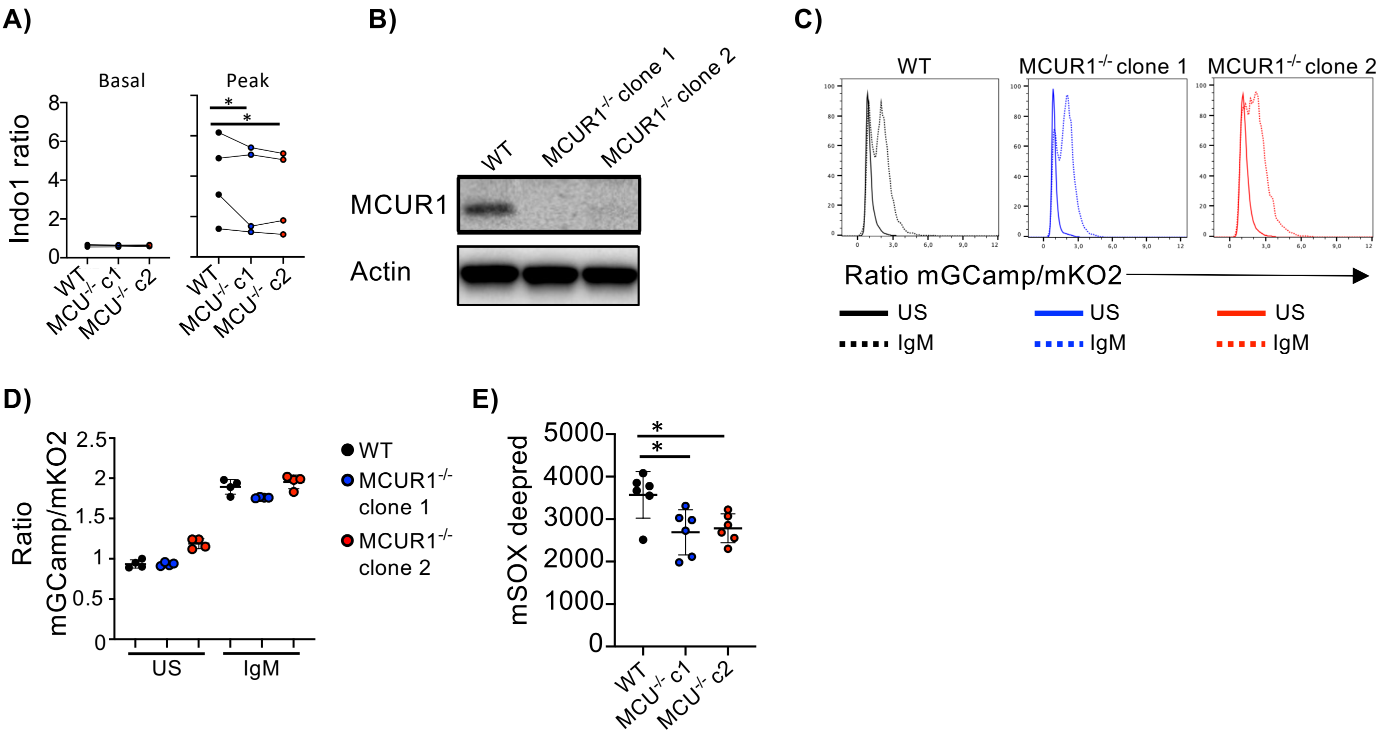


**Figure S5: MCUR1 is not needed for mCa$^{2+}$ uptake after BCR stimulation**

(A) Shown are the basal and the peak values of the Indo1-ratio from experiments performed as shown in Fig.5C. Shown results were obtained from 4 independent experiments. Significance was determined using the paired t-test. (B) Representative immunoblot analysis of MCUR1 protein expression in wild-type Ramos B cells compared to two MCUR1-deficient clones. Actin is used as a loading control. Representative of 2 independent experiments. (C) Mitochondrial Ca$^{2+}$ levels in wild-type and MCUR1-deficient Ramos cells expressing mGCaMP6s_mKO2 stimulated with anti- IgM (5µg/ml) for 1 min. The ratio of mGCaMP6s signal to mKO2 signal represents mCa$^{2+}$ levels. (D) Summary of mCa$^{2+}$ levels shown in Fig. S5C. (n=4 from 2 independent experiments) (E) Mitochondrial ROS levels of wild-type and MCU-deficient Ramos cells with expressing mGCaMP6s_mKO2 loaded with mtSOX deep red for 30 minutes. Pooled data from 6 independent experiments. In this plot, data are included that are also shown in Fig.5F. Data are presented as mean. ANOVA (E) was used to compare groups. *= $p < 0.05$.

Fig.S6.:

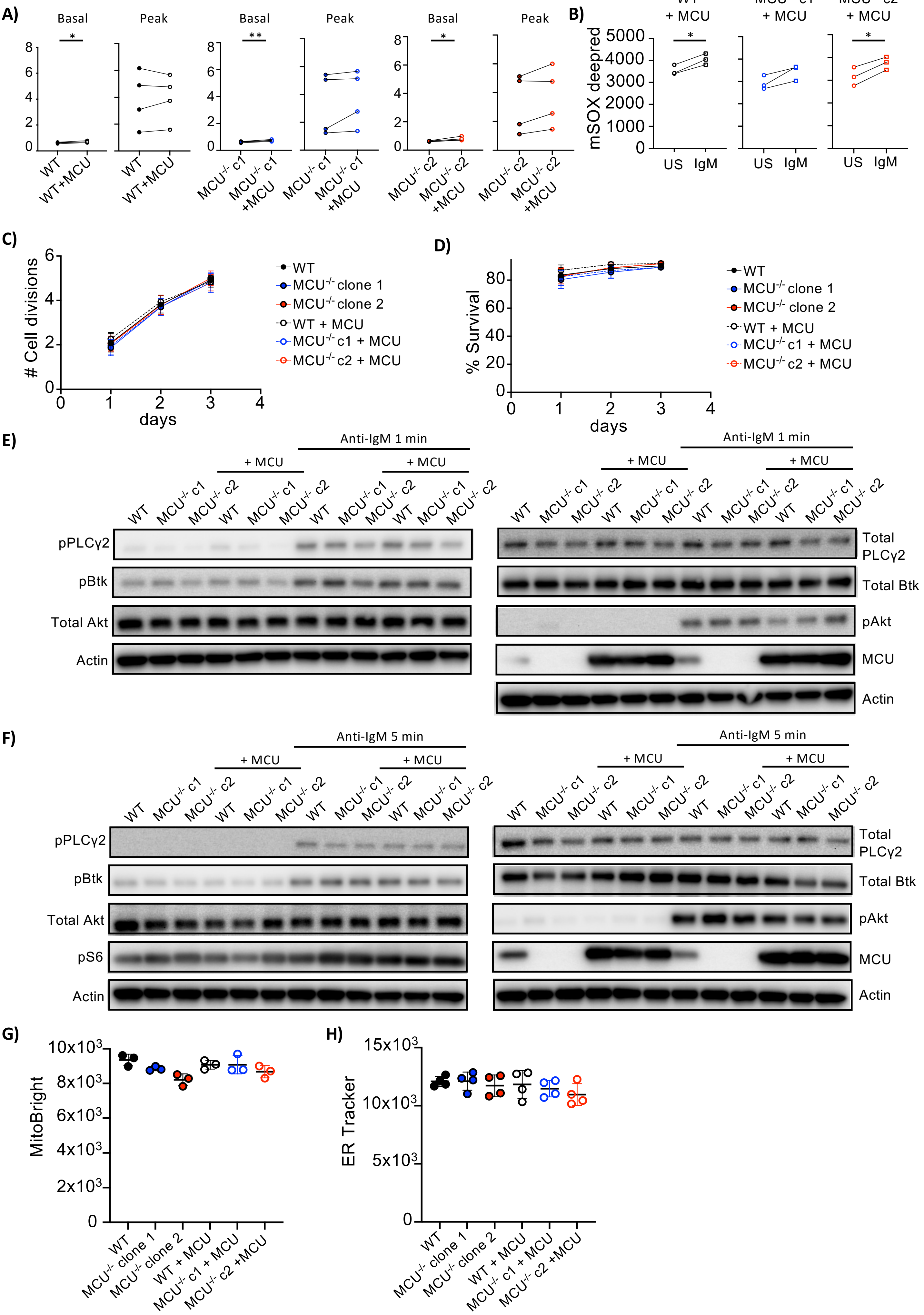
A)
Basal
Peak
WT
WT+MCU
MCU-/- c1
MCU-/- c1 +MCU
MCU-/- c2
MCU-/- c2 +MCU
B)
WT + MCU
MCU-/- c1 + MCU
MCU-/- c2 + MCU
mSOX deepred
US
IgM
C)
# Cell divisions
days
WT
MCU-/- clone 1
MCU-/- clone 2
WT + MCU
MCU-/- c1 + MCU
MCU-/- c2 + MCU
D)
% Survival
days
E)
Anti-IgM 1 min
+ MCU
pPLCγ2
pBtk
Total Akt
Actin
Total PLCγ2
Total Btk
pAkt
MCU
F)
Anti-IgM 5 min
pS6
G)
MitoBright
H)
ER Tracker

**Figure S6: Mitochondrial $Ca^{2+}$ overload does not affect B cell survival**

(A) Shown are the basal and the peak values of the Indo1-ratio from experiments performed as shown in Fig.6D. Shown results were obtained from 4 independent experiments. Significance was determined using the paired t-test. (B) MCU overexpressing WT Ramos cells and MCU deficient clones that were transduced with MCU were loaded with mtSOX deep red for 30 min and stimulated with anti- IgM (5µg/ml) for 1 min. Pooled data from 3 independent experiments. Data are presented as mean. Statistical significance was determined using the paired t-test. Results shown in A+B are from experiments that are also shown in Fig.S5A and Fig.5F respectively. (C) Average cell division of Ramos B cells calculated from eFluor 670 dilution after the indicated days. Pooled data from 4 independent experiments. (D) Survival of Ramos cells shown in Fig. S6C. Forward scatter (FSC) and side scatter (SSC) properties of the cells were used to determine the percentage of living cells. Pooled data from 4 independent experiments. (E+F) Control, MCU-deficient and MCU-overexpressing Ramos cells were stimulated with anti-IgM for 1min (E) and 5min (F). Lysates were loaded on two gels which were run in parallel to assess total and phospho-protein levels. Equal loading was verified by probing for actin. One of two independent experiments is shown.

(G) Summary of mitochondrial mass in wild-type, MCU-deficient and MCU-overexpressing Ramos cells expressing mGCaMP6s_mKO2 loaded with MitoBright LT deep red. Pooled data from 3 independent experiments. (H) Summary of ER mass levels in cells as described in Fig.S6C loaded with ER-Tracker Blue-White DPX. Pooled data from 4 independent experiments.

Fig.S7.:

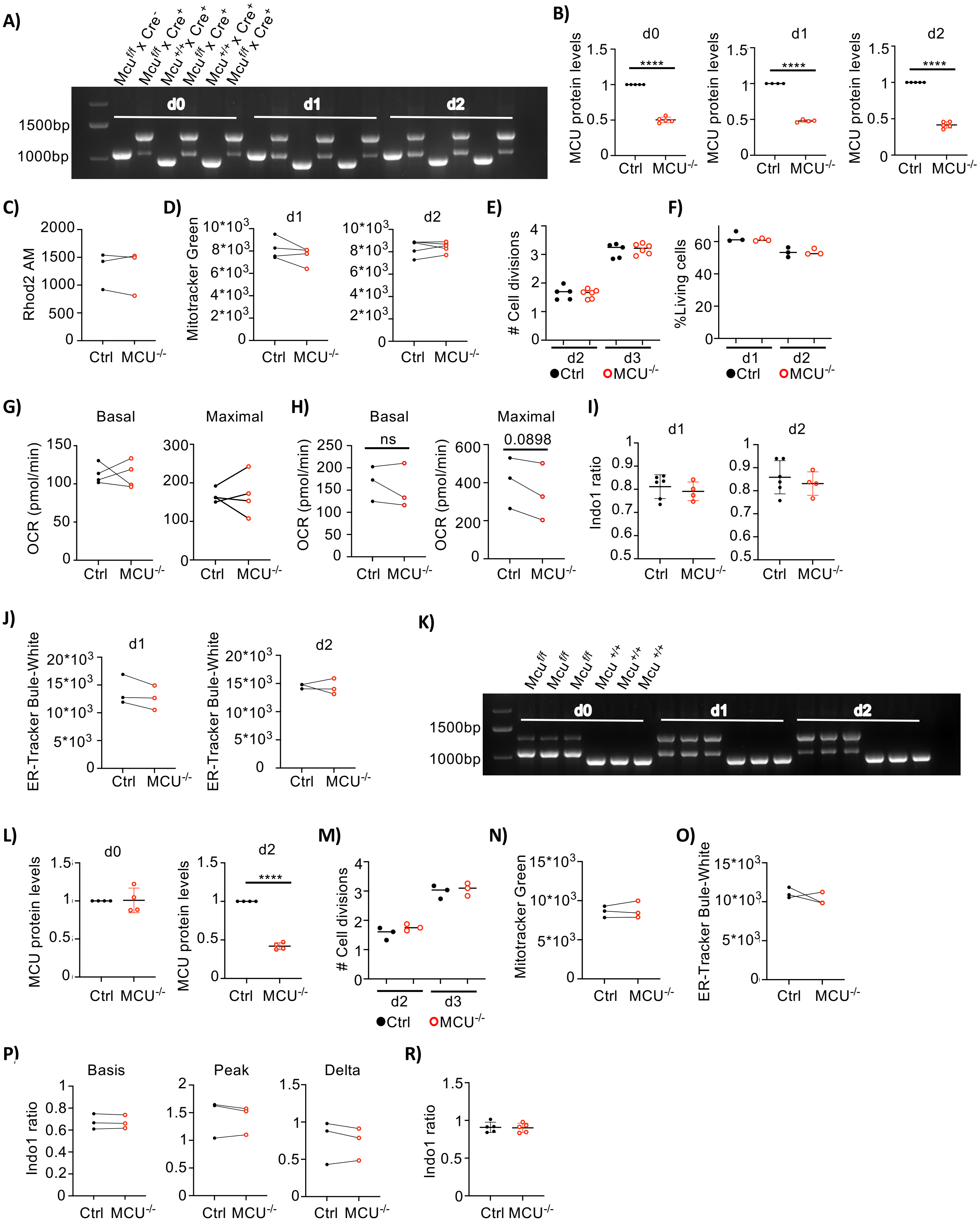
A)
Mcu f/f x Cre-
Mcu f/f x Cre+
Mcu +/+ x Cre+
Mcu f/f x Cre+
Mcu +/+ x Cre+
Mcu f/f x Cre+
d0
d1
d2
1500bp
1000bp
B)
d0
d1
d2
MCU protein levels
1.5
1
0.5
0
****
Ctrl
MCU-/-
C)
Rhod2 AM
2000
1500
1000
500
0
D)
Mitotracker Green
10*10^3
8*10^3
6*10^3
4*10^3
2*10^3
E)
# Cell divisions
●Ctrl
OMCU-/-
F)
%Living cells
60
40
20
G)
Basal
Maximal
OCR (pmol/min)
150
100
50
300
200
H)
250
200
150
100
50
ns
600
400
200
0.0898
I)
Indo1 ratio
1
0.9
0.8
0.7
0.6
0.5
J)
ER-Tracker Bule-White
20*10^3
15*10^3
10*10^3
5*10^3
K)
Mcu f/f
Mcu f/f
Mcu f/f
Mcu +/+
Mcu +/+
Mcu +/+
L)
M)
N)
15*10^3
10*10^3
5*10^3
O)
P)
Basis
Peak
Delta
0.8
0.6
0.4
0.2
2
1.5
R)

**Figure S7: B cells can compensate over time for the loss of MCU**

(A) To estimate *Mcu* gene deletion efficiency primers were designed which detect the Mcu-wt- (952 bp), Mcu-fl- (~1000 bp) allele and the deleted Mcu-fl-allele (~1250 bp). Three mice were treated with tamoxifen, B cells were purified and stimulated with anti-IgM. *Mcu* gene deletion was assessed at the indicated time points. (B) Mice were treated with tamoxifen, B cells were purified and stimulated with anti-IgM. MCU protein levels were assessed via westernblot at the indicated time points. An example experiment is shown in Fig.7A. Shown are values normalised to actin. Statistical significance was determined using one sample t test. Pooled data from 5 (for d0 and d2) and 4 (for d1) independent experiments. (d1: n=6 for $MCU^{-/-}$ and Ctrl mice; d0 and d2: n=7 for $MCU^{-/-}$ and Ctrl mice. (C) Mice were treated with tamoxifen, B cells were purified, $mCa^{2+}$ levels were assessed using Rhod-2AM. Shown are mean values from 3 independent experiments with a total of n= 5 for $MCU^{-/-}$ and Ctrl mice. (D) Mitochondrial mass (MitoTracker green FM staining) of B cells from Tamoxifen-injected $Mcu^{fl/fl}$ x $mb1^{CreERT2}$ ($MCU^{-/-}$) and control (Ctrl) mice stimulated for 1 (left) and 2 days (right) with anti-IgM. Pooled data from 4 and 5 independent experiments, respectively. (d1: n= 6 for $MCU^{-/-}$ and Ctrl mice, d2: n=7 $MCU^{-/-}$ mice 8 Ctrl mice). (E) Average cell division of mouse B cells shown in Fig. 7C. Pooled data from 3 independent experiments. (n=6 $MCU^{-/-}$ mice, 5 Ctrl mice). (F) Mice were treated with tamoxifen, B cells were isolated and stimulated with anti-IgM. Survival was assessed by Annexin + PI staining. Shown is the percentage of Annexin/PI negative cells. (G,H) Basal and maximal respiration in $MCU^{-/-}$ and Ctrl mouse B cells stimulated with anti-IgM as in Fig. 7A for 1 (G) and 2 days (H). Oxygen consumption rates (OCR) were measured using Seahorse flux technology as in Fig. 7M. Pooled data from 4 (d1) and 3 (d2) independent experiments. (d1: n=7 for $MCU^{-/-}$ and 6 for Ctrl; d2: n=5 for $MCU^{-/-}$ and 4 for Ctrl). (I) Cytosolic $Ca^{2+}$ levels stained with indo-1 AM in $MCU^{-/-}$ and Ctrl mouse B cells stimulated with anti-IgM as in Fig. 7A for 1 (left) and 2 days (right). Pooled data from 3 independent experiments. (n=4 for $MCU^{-/-}$ and 6 for Ctrl). (J) ER mass detected with ER-Tracker Blue-White DPX in $MCU^{-/-}$ and Ctrl mouse B cells stimulated with anti-IgM as in Fig. 7A for 1 (left) and 2 days (right). Pooled data from 3 independent experiments. (d1: n=3 for $MCU^{-/-}$ and Ctrl, d2: n=4 for $MCU^{-/-}$ and Ctrl). (K) B cells were treated with TAT-Cre and stimulated with anti-IgM. *Mcu*-gene deletion was assessed as shown in A). (L) B cells were purified, treated with TAT-Cre and stimulated with anti-IgM. MCU protein levels were assessed via westernblot at the indicated time points. An example experiment is shown in Fig.7H. Shown are values normalised to actin. Statistical significance was determined using one sample t test. (M) Average cell division of mouse B cells shown in Fig. 7J. Pooled data from 3 independent experiments. (n=3 for $MCU^{-/-}$ and Ctrl mice). (N) Mitochondrial mass (MitoTracker green FM) of $MCU^{-/-}$ and Ctrl mouse B cells stimulated with anti-IgM as in Fig. 7H for 2 days. Pooled data from 3 independent experiments. (n=4 for $MCU^{-/-}$ and Ctrl mice). (O) ER mass (ER-Tracker Blue-White DPX ) of $MCU^{-/-}$ and Ctrl mouse B cells stimulated as in Fig. 7H for 2 days. Pooled data from 3 independent experiments. (n=4 for $MCU^{-/-}$ and Ctrl mice). (P) Cytosolic $Ca^{2+}$ was assessed in TAT-CRE treated $Mcu^{fl/fl}$ and Ctrl B cells using indo-1 AM. Shown are basal and peak $Ca^{2+}$ levels after anti-IgM stimulation as well as the difference between peak and basal levels (delta). Pooled data from 3 independent experiments. (n=5 for $MCU^{-/-}$ and Ctrl mice). (R) TAT-CRE treated $Mcu^{fl/fl}$ and Ctrl B cells were stimulated with anti-IgM. Cytosolic $Ca^{2+}$ levels were assessed after 2 days of culture using indo-1 AM. Pooled data from 3 independent experiments. (n=5 for $MCU^{-/-}$ and Ctrl mice). Data are presented as mean. Paired and unpaired Student's t-test was used to compare groups. ****= $p < 0.0001$, ns= not significant.
